# Sunlight PDT leveraging NIR-II nanospray: painless, hemostatic, anti-inflammatory therapy towards diabetic wound infections

**DOI:** 10.1093/nsr/nwaf554

**Published:** 2025-12-05

**Authors:** Qihang Ding, Lingbo Zhou, Tao Xiong, Jiqiang Liu, Luojia Chen, Jiyoung Yoo, Xiaoyu Xu, Xianglei Jia, Siling Chen, Siyu Chen, Yifan Wang, Ping Gong, Meijia Gu, Wen Jiang, Jong Seung Kim

**Affiliations:** Department of Chemistry, Korea University, Seoul 02841, Republic of Korea; Department of Neurosurgery, Zhongnan Hospital of Wuhan University, Ministry of Education Key Laboratory of Combinatorial Biosynthesis and Drug Discovery, School of Pharmaceutical Sciences, Wuhan University, Wuhan 430071, China; Renmin Hospital of Wuhan University, Wuhan 430060, China; College of Chemistry and Chemical Engineering, Central South University, Changsha 410083, China; Guangdong Key Laboratory of Nanomedicine, Chinese Academy of Sciences-Hong Kong Joint Lab for Biomaterials, Chinese Academy of Sciences Key Laboratory of Biomedical Imaging Science and System, Center for Nanomedicine and Nanobiotechnology, Institute of Biomedicine and Biotechnology, Shenzhen Institutes of Advanced Technology, Chinese Academy of Sciences, Shenzhen 518055, China; Department of Neurosurgery, Zhongnan Hospital of Wuhan University, Ministry of Education Key Laboratory of Combinatorial Biosynthesis and Drug Discovery, School of Pharmaceutical Sciences, Wuhan University, Wuhan 430071, China; Department of Chemistry, Korea University, Seoul 02841, Republic of Korea; Department of Neurosurgery, Zhongnan Hospital of Wuhan University, Ministry of Education Key Laboratory of Combinatorial Biosynthesis and Drug Discovery, School of Pharmaceutical Sciences, Wuhan University, Wuhan 430071, China; Renmin Hospital of Wuhan University, Wuhan 430060, China; Department of Neurosurgery, Zhongnan Hospital of Wuhan University, Ministry of Education Key Laboratory of Combinatorial Biosynthesis and Drug Discovery, School of Pharmaceutical Sciences, Wuhan University, Wuhan 430071, China; Department of Neurosurgery, Zhongnan Hospital of Wuhan University, Ministry of Education Key Laboratory of Combinatorial Biosynthesis and Drug Discovery, School of Pharmaceutical Sciences, Wuhan University, Wuhan 430071, China; Department of Radiation Oncology, The University of Texas MD Anderson Cancer Center, Houston, TX 77030, USA; Guangdong Key Laboratory of Nanomedicine, Chinese Academy of Sciences-Hong Kong Joint Lab for Biomaterials, Chinese Academy of Sciences Key Laboratory of Biomedical Imaging Science and System, Center for Nanomedicine and Nanobiotechnology, Institute of Biomedicine and Biotechnology, Shenzhen Institutes of Advanced Technology, Chinese Academy of Sciences, Shenzhen 518055, China; Department of Neurosurgery, Zhongnan Hospital of Wuhan University, Ministry of Education Key Laboratory of Combinatorial Biosynthesis and Drug Discovery, School of Pharmaceutical Sciences, Wuhan University, Wuhan 430071, China; Department of Radiation Oncology, The University of Texas MD Anderson Cancer Center, Houston, TX 77030, USA; Department of Chemistry, Korea University, Seoul 02841, Republic of Korea

**Keywords:** sunlight, photodynamic therapy, antibacterial, reactive oxygen species, NIR-II

## Abstract

Photodynamic therapy (PDT), which relies on the activation of photosensitizers by specific wavelengths of light to generate reactive oxygen species (ROS) for targeted pathogen or diseased tissue eradication, offers substantial promise for clinical wound management. However, its application in diabetic wound management remains constrained by suboptimal therapeutic efficacy, recurrent infections, treatment-associated pain, scar formation, and dependence on costly specialized equipment. Here, we present a sunlight-activated nanospray formulation comprising chitosan oligosaccharide-coated nanoparticles (SPS), engineered for the rapid and effective management of diabetic wounds in outdoor and resource-limited settings. SPS enables efficient ROS generation under ambient natural light, thereby reducing treatment-associated discomfort while maintaining effective photodynamic antimicrobial action. Additionally, the chitosan oligosaccharide coating confers intrinsic hemostatic, antibacterial, and antioxidative properties that synergistically accelerate wound closure and reduce the risk of scarring. The topical spray delivery circumvents systemic phototoxicity, improving patient compliance and broadening the accessibility of PDT. This strategy effectively overcomes the inherent limitations of conventional PDT by leveraging ambient light for activation and providing a cost-effective, non-invasive, and patient-friendly platform for diabetic wound care, thereby expanding the clinical utility of photodynamic interventions for managing chronic and complex wounds.

## Teaser

A sunlight-activated nanospray provides a painless, equipment-free way to kill bacteria and speed diabetic wound healing, offering an accessible solution for infection control in everyday settings.

## INTRODUCTION

Photodynamic therapy (PDT) is a therapeutic approach that combines photosensitizers with specific wavelengths of light to treat various diseases [1,2]. The fundamental mechanism involves the activation of a photosensitizer by light energy, leading to the generation of reactive oxygen species (ROS), which subsequently induce cytotoxic effects on target cells or pathogens [3–6]. While PDT has shown promise in treating cancers, dermatological disorders, and certain infectious diseases, its clinical translation remains limited due to several critical drawbacks [7–12]. First, the limited penetration depth of light presents a significant challenge. Visible light used in PDT has restricted tissue penetration, making it difficult to effectively treat deep-seated lesions. Second, systemic phototoxicity remains a major concern. Photosensitizers can exert toxic effects on surrounding healthy cells upon light exposure, necessitating prolonged post-treatment light avoidance to prevent severe skin phototoxicity. Third, PDT is often associated with treatment-induced pain and inflammation. The commonly used laser-based PDT light sources generate intense localized heat, causing discomfort at the treatment site, as well as inducing inflammation and erythema in adjacent healthy

tissues [13–16]. These adverse effects significantly impact patient compliance and overall treatment tolerance. Lastly, PDT requires complex and costly equipment. The precise control of light wavelength, intensity, and exposure duration is essential for efficacy, necessitating expensive and sophisticated laser, light-emitting diode (LED), or fiber-optic systems. Longer-wavelength light, such as near-infrared (NIR), offers deeper tissue penetration but requires more advanced instrumentation. Additionally, maintaining homogeneous and adequate light intensity is critical for therapeutic outcomes, further increasing technical complexity [17–22]. The bulkiness and non-portability of PDT devices, along with the need for specialized training, further hinder its widespread clinical adoption.

With the global prevalence of diabetes rising [23–25], impaired wound healing in diabetic patients has become a pressing medical challenge, severely compromising patient health and quality of life while imposing a substantial socioeconomic burden. Diabetic wounds exhibit significantly delayed healing due to hyperglycemia-induced metabolic dysfunction, reduced cellular activity, vascular impairment, extracellular matrix degradation, and chronic inflammation [26–30]. These pathological conditions disrupt the natural wound-healing cascade, exacerbating systemic complications and increasing susceptibility to infections. Given the compromised immune response, increased infection risk, and impaired angiogenesis in diabetic patients, developing more effective therapeutic strategies is imperative. Ideal treatments should accelerate wound healing, enhance vascularization, mitigate infection risks, and enable continuous wound monitoring and management [31–33].

**Scheme 1.**
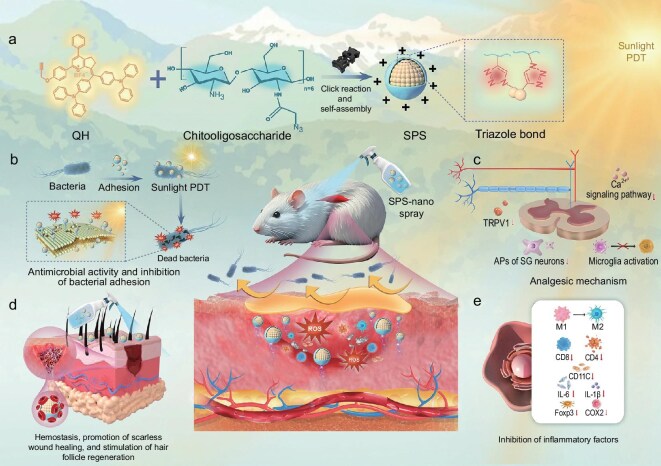
Construction of SPS-nanospray (a) and its application in sunlight-activated photodynamic therapy for wound healing (b), painless treatment (c), hemostasis (d), and anti-inflammation (e) in diabetic wound infections.

In this study, we synthesized a novel photosensitizer, designated as QH, and subsequently fabricated nanoparticles, termed SPS, by self-assembly with chitosan oligosaccharides via an alkyne–azide click reaction. Compared with hydrogels or drops, sprays offer more uniform coverage, greater comfort, controllable dosing, and faster absorption, while also providing better hygiene and portability, making them more suitable for clinical and outdoor applications. Therefore, we further developed these nanoparticles into a spray formulation for emergency management of diabetic wounds in outdoor settings. The SPS exhibits a broad ROS excitation spectrum, spanning from the ultraviolet (UV) to visible light range, and possesses exceptional ROS-generating efficiency. Even under low-intensity light, SPS efficiently produces substantial ROS levels, ensuring robust antimicrobial activity at the wound site. To enhance practicality and minimize patient discomfort, we innovatively employed natural sunlight as the excitation source, offering a cost-effective, accessible, and convenient alternative to traditional laser-based PDT [34–37]. Given the high ROS generation efficiency of SPS, the variability and low intensity of natural sunlight do not compromise therapeutic efficacy. Instead, the use of ambient natural sunlight alleviates the pain and discomfort associated with high-intensity laser irradiation, thereby improving patient compliance. Furthermore, the chitosan oligosaccharide coating of SPS exhibits excellent hemostatic properties. As a highly water-soluble, positively charged, and non-immunogenic material, it not only provides antibacterial and antioxidant effects at the wound site but also suppresses excessive inflammatory responses, thereby promoting scarless wound healing [38–40]. Lastly, the topical spray administration effectively circumvents the systemic phototoxicity commonly associated with conventional PDT, enhancing treatment safety and accessibility. In conclusion, we have developed a photosensitizer-based nanomaterial designed for emergency diabetic wound care, leveraging natural sunlight as an excitation source. This innovative approach addresses the key limitations of conventional photosensitizers, particularly in field-based and resource-limited settings, and represents a promising advancement in diabetic wound management (Scheme 1).

## RESULTS AND DISCUSSION

### Design, synthesis, and characterization of SPS

**Figure 1.**
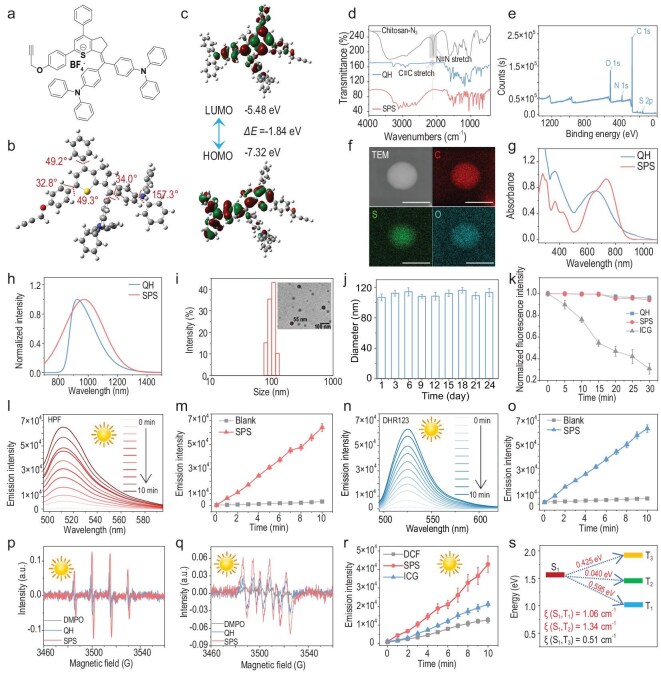
Nanoplatform construction and characterization. (a) Chemical structure of QH. (b) Computational results of the optimized molecular geometries. (c) Frontier molecular orbitals and energy levels (eV) obtained from DFT calculations. (d) FTIR spectra of chitosan oligosaccharides, QH, and SPS. (e) XPS measurement spectrum of SPS. (f) Elemental mapping of SPS showing distributions of C, S, and O elements (scale bars: 50 nm). (g) Absorption spectra and (h) emission spectra of QH and SPS in DCM and PBS buffer, respectively. (i) DLS size distribution and representative TEM image of SPS (scale bar: 100 nm). (j) Size stability of SPS (100 μM) in DMEM over 24 days. (k) Photostability of QH, SPS, and ICG in PBS buffer under continuous 808 nm laser irradiation (1.0 W/cm^2^), with fluorescence intensity recorded at predetermined time points (0, 5, 10, 15, 20, 25, and 30 min). (l–o) Relative changes in emission intensity of (l, m) HPF (515 nm) and (n, o) DHR123 (523 nm), in the presence of SPS (20 μM) upon sunlight irradiation (45.5 mW/cm^2^) for different durations. (p, q) EPR signals of DMPO in the presence of QH and SPS (20 μM) in PBS buffer, with (p) or without (q) sunlight irradiation (∼ 45.5 mW/cm^2^) for 5 min. (r) Relative changes in emission intensity of DCFH (525 nm) in the presence of SPS and ICG (20 μM) upon sunlight irradiation (45.5 mW/cm^2^) over time. (s) Calculated lowest excited singlet (S_1_) and triplet energy (T_1_–T_3_) energy levels of QH (CAM-B3LYP/6–31G(d) level) and the SOC constants between the lowest excited singlet (S_1_) and triplet energy level (T_1_–T_3_) of the QH.

We synthesized a novel second near-infrared window (NIR-II) organic small molecule, QH, following synthetic methods previously reported by our laboratory [41] (Fig. 1a). The compound was thoroughly characterized using ^1^H NMR, ^13^C NMR, and ESI-HRMS (Figs S1–S3). Density functional theory (DFT) calculations were subsequently performed using the 6–31G(d) basis set and B3LYP exchange-correlation functional to determine the highest occupied molecular orbital (HOMO) and the lowest unoccupied molecular orbital (LUMO) energy levels of QH. The computational results indicated that QH exhibits a HOMO energy of −7.32 eV and a LUMO energy of −5.48 eV, yielding a bandgap of 1.84 eV, which is characteristic of typical NIR-II optical energy gaps. Additionally, the optimized molecular structure of QH revealed a twisted conformation, with dihedral angles of 34.0° and 49.3° between the bis(4-(diphenylamino) phenyl) methanone and thiazolidine units (Fig. 1b and c). This conformation renders the molecule no longer completely coplanar but instead exhibits a typical donor-acceptor (D-A) twisted structure. On the one hand, the moderate distortion weakens the π-conjugation, which facilitates intramolecular charge transfer, thereby narrowing the bandgap and inducing a red shift in absorption/emission. On the other hand, the nonplanar configuration effectively suppresses strong intermolecular π–π stacking, reducing fluorescence quenching and thus enhancing its luminescent performance in the NIR region [42–44]. To enhance the water solubility and biocompatibility of QH, its alkynyl and azide groups were conjugated with shell oligosaccharides via a click reaction, leading to the formation of a self-assembled nanoparticle platform (SPS). Chitosan oligosaccharides were selected over chitosan due to their lower molecular weight, better solubility, and ability to dissolve directly in water. Additionally, oligosaccharides exhibit enhanced biological activity in antimicrobial, hemostatic, and anti-inflammatory applications, along with low toxicity and excellent biocompatibility [45]. The successful construction of SPS was confirmed through Fourier transform infrared (FTIR) spectroscopy (Fig. 1d), which revealed the disappearance of the azide band on chitosan oligosaccharides and the alkyne band on the photosensitizer QH after the formation of SPS, indicating successful conjugation. Moreover, high-resolution X-ray photoelectron spectroscopy (XPS) analysis of the sulfur (S) 2p region (Fig. 1e) further confirmed the successful self-assembly of QH with chitosan oligosaccharides to form SPS. Furthermore, elemental analysis using scanning transmission electron microscopy-energy dispersive spectroscopy (STEM-EDS) confirmed the presence of sulfur, a characteristic element of QH, verifying the successful modification of the oligosaccharides (Fig. 1f). QH displayed strong NIR optical characteristics, with a maximum absorption at 661 nm (10 μM) and an emission peak at 930 nm in dichloromethane (DCM), accompanied by a pronounced emission tail reaching into the NIR-II window (Fig. 1g and h). In contrast, SPS in deionized water exhibited an absorption peak at 736 nm and an emission peak at 970 nm, representing only a slight redshift compared to QH. This minor spectral shift suggests that oligosaccharide modification exerts minimal influence on the intrinsic photophysical properties of QH. The morphology and size distribution of SPS were further analyzed using transmission electron microscopy (TEM) and dynamic light scattering (DLS), revealing uniform spherical nanoparticles with an average diameter of 55 nm by TEM and 102 nm by DLS (Fig. 1i). The zeta potential of SPS was measured at +15.5 mV (Fig. S4), which, while indicating moderate colloidal stability that may lead to gradual aggregation during extended storage, is sufficient to promote electrostatic interactions with negatively charged bacterial membranes, thereby facilitating bacterial adhesion and subsequent membrane disruption for antimicrobial applications. Notably, SPS demonstrated colloidal stability under physiological conditions, maintaining a consistent hydrodynamic diameter for 24 days in Dulbecco’s Modified Eagle Medium (DMEM) containing 10% Fetal Bovine Serum (FBS) (Fig. 1j).

We also compared the photostability of QH and SPS with indocyanine green (ICG), the only U.S. Food and Drug Administration (FDA)-approved clinical imaging agent, under continuous excitation (1 W/cm^2^) for 30 min in phosphate-buffered saline (PBS) (Fig. 1k). The fluorescence intensity of SPS exhibited negligible decay, and the particle size, as measured by DLS, remained unchanged (Fig. S5). In contrast, ICG exhibited a significant reduction in brightness after 30 min of irradiation, highlighting the superior photostability of QH and SPS compared to ICG.

Next, we systematically evaluated the photodynamic properties of SPS. The generation of singlet oxygen (^1^O_2_) was measured using 1,3-diphenylisobenzofuran (DPBF) as a probe under 808 nm laser irradiation (1 W/cm^2^). The absorbance of DPBF in the SPS solution at 410 nm remained largely unchanged over 200 s, with a much smaller decrease than that observed in the DPBF solution containing ICG under identical conditions (Fig. S6a and c). These results indicate that SPS does not primarily generate ROS via the type II photochemical mechanism (Fig. S7).

To further investigate ROS generation pathways, we utilized dihydrorhodamine 123 (DHR123) to detect superoxide anions (O_2_⁻•) and hydroxyphenyl fluorescein (HPF) to detect hydroxyl radicals (•OH) under 808 nm laser irradiation at varying power densities (0.25 W/cm^2^ and 0.5 W/cm^2^). The results revealed that SPS predominantly generates O_2_⁻• and •OH via type I photochemical pathways (Figs S8–S11). Notably, under natural sunlight (solar irradiance measured at 45.5 mW/cm^2^ using a photo densitometer), SPS also generated substantial amounts of O_2_⁻• and •OH, mirroring the results obtained under 808 nm laser irradiation (Fig. 1l–o, Figs S12 and S13). Electron paramagnetic resonance (EPR) spectroscopy using 5,5-dimethyl-1-pyrroline-*N*-oxide (DMPO) further confirmed the generation of O_2_⁻• and •OH under sunlight exposure (Fig. 1p and q). Additionally, 2′,7′-dichlorodihydrofluorescein (DCFH) probe tests demonstrated that SPS produced significantly higher levels of ROS under sunlight compared to ICG (Fig. 1r).

To explore the potential of QH in PDT, we performed time-dependent density functional theory (TD-DFT) calculations at the PBE0/def2-TZVP level (Fig. 1s). These calculations examined the singlet and triplet excited states of QH to assess its potential for ROS generation. As shown in Fig. 1r, the energy gap between the lowest singlet excited state (S_1_) and the triplet excited state (T_2_) was only 0.04 eV, providing favorable conditions for intersystem crossing (ISC). Further calculations indicated that QH exhibits strong spin-orbit coupling (SOC) of 1.34 cm⁻^1^ between S_1_ and T_2_, facilitating efficient spin transitions that enhance PDT efficacy. Collectively, these results underscore the potential of QH for PDT, revealing its advantageous electronic structure and excited-state dynamics that enable efficient ROS generation, which is crucial for its application in subsequent sunlight-guided PDT.

Finally, we evaluated the photothermal conversion capability of SPS. Under 808 nm laser irradiation (1.0 W/cm^2^), SPS exhibited relatively weak photothermal conversion performance (Fig. S14). Notably, excessive photothermal effects can lead to non-specific heating of surrounding tissues or the environment, potentially causing unintended damage to healthy tissues or cells. Overheating in the target area may result in irreversible tissue damage and trigger uncontrolled inflammation.

### SPS exhibits potent bactericidal effect *in vitro*

**Figure 2.**
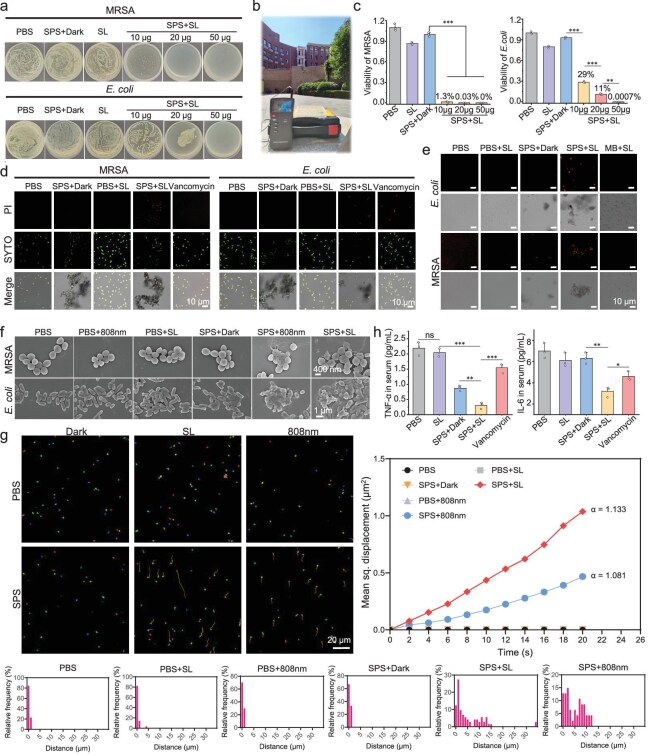
*In vitro* assay with SPS on common bacteria and its comparison with vancomycin. (a) Colony growth after MRSA and *E. coli* coated plates were treated under different conditions (sunlight, denoted as SL). (b) Real-life image of solar illumination; light condition: 50 mW/cm^2^ sunlight at 2 p.m. (c) Statistical graph of bacterial killing experiments (*n* = 3, *P < 0.05, **P < 0.01, ***P < 0.001, n.s.: not statistically significant, one-way Analysis of Variance (ANOVA)). (d) Live and dead MRSA and *E. coli* detected using the SYTO 9-PI staining method after treatment under different conditions (scale bars: 10 μm). (e) Confocal microscopy images of MRSA and *E. coli* after treatment under different conditions (MB in the figure denotes methylene blue, scale bars: 10 μm). (f) SEM images of treated bacterial samples (scale bars: 400 nm (top), 1.0 μm (bottom)). (g) CLSM images of *E. coli* movement under different conditions (scale bar: 20 μm). (h) ELISA immunoassay of pro-inflammatory factors TNF-α and IL-6 in different treatment groups.

We first evaluated the antimicrobial efficacy of SPS *in vitro* against common bacterial strains, including methicillin-resistant *Staphylococcus aureus* (MRSA) and *Escherichia coli* (*E. coli*). Bacteria were subjected to different treatment conditions: phosphate-buffered saline (PBS, control), SPS in the absence of light (SPS + dark), and SPS under sunlight irradiation (SPS + sunlight). SPS was administered at concentrations of 10, 20, and 50 μg (Fig. 2a). For light-treated groups, samples were exposed to sunlight at an intensity of ∼50 mW/cm^2^ (Fig. 2b) for 10 min. Following treatment, bacterial suspensions were spread onto agar plates, and control groups (PBS and SPS + dark) were incubated under identical conditions without light exposure. All treatment groups were incubated under identical conditions overnight at 37°C. Post-incubation bacterial growth assessment revealed a significant reduction in bacterial survival in the SPS + sunlight groups, indicating a strong photodynamic bactericidal effect. To confirm these findings, bacterial viability was quantified (Fig. 2c, left), demonstrating a significantly lower survival rate in the SPS + sunlight groups compared to the SPS + dark and PBS-treated groups. The bactericidal effect was further evaluated using SYTO 9 green fluorescent nucleic acid stain and propidium iodide (PI) staining, where live bacteria (green fluorescence) were observed under a fluorescein filter (Fig. 2d), and dead bacteria (red fluorescence) were observed under a Texas Red filter (Fig. 2e). Statistical analysis (Fig. 2c, right) confirmed that bacterial survival following SPS (50 μg/mL) + sunlight treatment was significantly lower than that in the dark group or the PBS-treated group and was even lower than that of the vancomycin (50 μg/mL)-treated positive control group. Additionally, electron microscopy imaging was performed on MRSA and *E. coli* subjected to different treatment conditions. Bacterial membrane rupture was observed following SPS treatment under both 808 nm laser irradiation and sunlight exposure. In contrast, bacteria treated with PBS alone, PBS + 808 nm, PBS + sunlight, or SPS + dark retained normal cellular structures. Scanning electron microscopy (SEM) images (Fig. 2f) clearly depict bacterial rupture and structural damage upon treatment with SPS in combination with sunlight or 808 nm laser irradiation. In conclusion, SPS exhibited potent antibacterial activity under both sunlight and 808 nm NIR light, demonstrating a bactericidal effect superior to that of conventional antibiotics. Bacterial adhesion is the initial step in biofilm formation [46]. Therefore, preventing adhesion can effectively inhibit biofilm development at its source. In addition, we have added the evaluation of SPS antibacterial effects against *E. coli* under conditions of insufficient light on cloudy days (10 mW/cm^2^) and an alternative smartphone lighting setup indoors (∼45 mW/cm^2^) (Fig. S15). These findings further support the robustness of SPS activation under variable lighting conditions.

To assess the impact of different treatments on bacterial adhesion, we tracked the movement trajectories of *E. coli* using a confocal laser scanning microscope (CLSM). Following treatment, different groups of *E. coli* were diluted to the same concentration, labelled with NucGreen for visualization, and their motility was recorded using time-lapse live confocal microscopy for 20 s, capturing 11 frames at 2 s intervals. Bacterial adhesion was evaluated by analyzing the videos using the tracking plug-in for NIS-Elements. Individual bacterial movements were tracked, relevant motility parameters were calculated, and movement trajectories were converted into images (Fig. 2g). Time-averaged mean squared displacement (MSD) is commonly used to quantify micromaterial motion and was first applied in tracking genetic locus trajectories within the nucleus [47]. MSD represents the average of squared displacements of an observed locus over different time intervals, calculated as MSD(Δt) = ⟨[x(t+Δt)−x(t)]^2^⟩. The population MSD (pMSD) is obtained by averaging MSD values across bacteria and plotting them on a log-log scale. The slope of this plot determines the anomalous exponent α, also known as the scaling or diffusion exponent, which classifies bacterial motion into four categories [48]: subdiffusion (α <1), superdiffusion or directed motion (α >1), pure Brownian motion (α = 1), and ballistic motion (α = 2). Thus, MSD analysis provides critical insights into bacterial motility, including movement range, velocity, and motion type. The α values for each treatment group were calculated, revealing that SPS + PDT treatment resulted in the highest α values among all conditions (control + dark: 0.0582; control + sunlight: 0.0231; control + 808 nm: 0.0532; SPS + dark: 0.0350; SPS + sunlight: 1.1330; SPS + 808 nm: 1.0812). This indicates that *E. coli* exhibited the highest motility following SPS + sunlight treatment, correlating with the weakest adhesion ability. Additionally, the proportion of bacteria displaying increased displacement was also significantly higher in this group. These findings suggest that SPS + sunlight treatment strongly enhances bacterial motility while inhibiting bacterial adhesion, thereby preventing biofilm formation.

To assess variations in the inflammation response, we used enzyme-linked immunosorbent assay (ELISA) immunoassay to quantify serum levels of tumor necrosis factor-alpha (TNF-α) and interleukin-6 (IL-6) (Fig. 2h). Both inflammatory markers were significantly reduced in the SPS + sunlight and vancomycin treatment groups compared to those treated with PBS alone, sunlight alone, or SPS + dark. Notably, the SPS + sunlight group exhibited the lowest serum levels of TNF-α and IL-6, indicating that SPS provides the strongest anti-inflammatory effect. Additionally, to assess the toxic effects of SPS on normal cells, we conducted hemolysis experiments on blood of mice (Fig. S16) and Cell Counting Kit-8 (CCK-8) assay on human embryonic kidney 293 (HEK293) cells (Fig. S17). The results demonstrated that as the concentration of SPS increased (up to 100 μg/mL), there was no significant cell death observed in normal cells, nor was there noticeable hemolysis in blood cells, demonstrating the biosafety of SPS.

**Figure 3.**
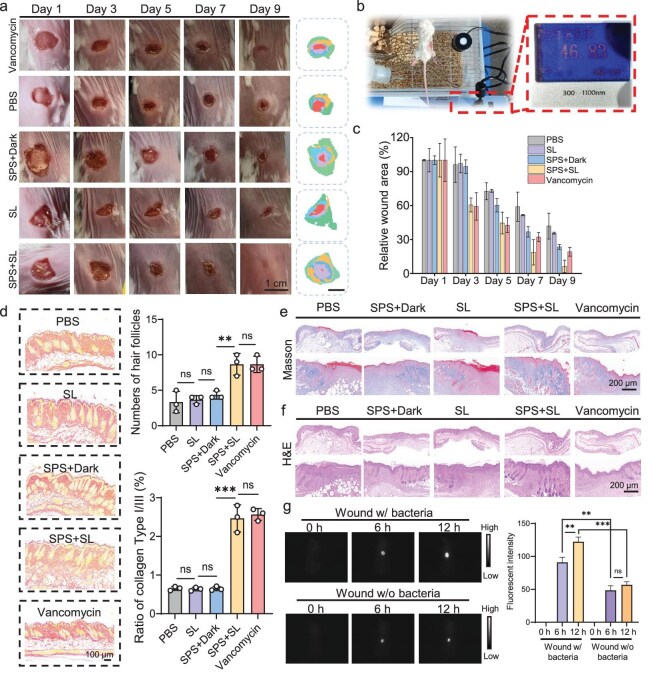
Wound healing effects on different treatment methods. (a) Changes in wound size across five treatment groups over 9 days (scale bar: 1 cm). (b) Representative image of sunlight-treated wounds in a mouse. (c) Wound healing statistics for different treatment groups. (d) Representative images of Sirius red staining for hair follicle analysis (scale bar: 100 μm). Right: statistical analysis of hair follicle count and collagen I/III ratio in wound-healed skin across different groups (*n* = 3 per group). (e and f) Masson and H&E staining of different groups (scale bars: 200 μm). (g) NIR imaging of mouse wounds; from left to right: fluorescence pseudo-color images of mouse wounds irradiated with 808 nm NIR light before dosing, and at 6 h and 12 h post-dosing (*n* = 3 per group). Right: statistical analysis of standardized fluorescence intensity in NIR imaging of mouse wounds. *P < 0.05, **P < 0.01, ***P < 0.001, n.s. (not statistically significant, one-way ANOVA).

SPS promotes wound repair and regeneration *in vivo*. To evaluate the *in vivo* therapeutic efficacy of SPS, we applied the nanospray to mouse wounds and monitored the wound healing process. Prior to wound incision, all mice received intraperitoneal injections of streptozotocin (STZ) until blood glucose levels stabilized above the standard range (Fig. S18a). Body weight was monitored simultaneously (Fig. S18b). Wounds (diameter ∼1.0 cm) were treated with vancomycin, PBS, SPS + dark, sunlight, or SPS + sunlight. Representative wound images captured at predetermined time intervals are shown in Fig. 3a, with pseudo-color wound healing images displayed on the right [49]. Regardless of the treatment method, all wounds exhibited size reduction by the day 9. However, wounds treated with SPS + sunlight healed the fastest, leaving only faint granulation tissue by the day 9. A schematic diagram of the sunlight-treated mouse wounds (50 mW/cm^2^) is shown in Fig. 3b. The statistical chart (Fig. 3c) shows that wound contraction was observed in all five groups over 9 days, with the SPS + sunlight group exhibiting the most significant reduction in wound size. Collagen deposition and hair follicle alterations are key indicators of the wound healing process [50]. To investigate these changes, we employed Picrosirius red staining, a technique known for its high collagen-binding affinity and specificity, to differentiate collagen subtypes. This method enables the visualization of type I collagen, which predominates in healthy dermal tissue, and type III collagen, which is abundant in scar formations. Additionally, Picrosirius red staining facilitated the visualization of hair follicle morphology in regenerating skin [51]. By 1 week post-treatment, hair follicles near the wound margins were clearly visible in the SPS + sunlight group. Quantitative analysis revealed a significant increase in hair follicle density and enhanced collagen organization in the treatment group compared to the control (Fig. 3d). These findings demonstrate that SPS (50 μg/mL) significantly accelerates wound healing under sunlight exposure, achieving therapeutic outcomes comparable to those observed with vancomycin (300 μg/mL) administration.

Histological analyses of periwound tissue were subsequently performed using Masson’s trichrome and Hematoxylin & Eosin (H&E) staining to assess the effects of different treatments on wound healing (Fig. 3e and f). By the day 7, tissue specimens from the SPS-treated group exhibited pronounced re-epithelialization at the wound centre, indicating accelerated tissue regeneration. In contrast, control group samples displayed limited neotissue formation, characterized by extensive localized hemorrhage and a disorganized, loosely arranged connective tissue architecture, indicative of impaired healing dynamics.

To further evaluate the *in vivo* antimicrobial efficacy of SPS, we conducted fluorescence imaging to assess its activity against biofilm-associated infections. NIR-II imaging was employed to monitor the fluorescence intensity of wounds before and after SPS administration (Fig. 3g). At baseline (0 h), no detectable fluorescence was observed in wounds, irrespective of bacteria presence. However, a distinct fluorescence signal emerged 6 h post-administration, with bacteria-containing wounds exhibiting significantly stronger fluorescence than those without bacteria. This trend persisted at 12 h post-treatment, indicating a sustained response in bacteria-associated wounds. These findings suggest that SPS demonstrates effective *in vivo* antimicrobial activity, particularly in bacteria-infected environments.

### SPS treatment decreases inflammatory responses in skin wounds

**Figure 4.**
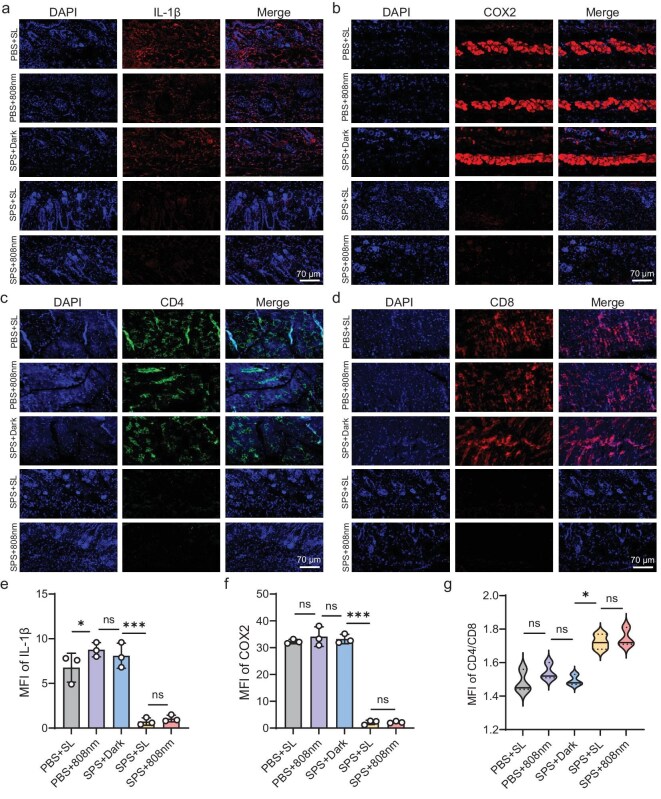
Decreasing inflammatory response upon sunlight PDT. (a–d) Representative immunofluorescence images of IL-1β, COX-2, CD4, and CD8 in skin tissue across different subgroups, with DAPI co-labeling (scale bars: 70 μm). (e) Quantitative analysis of mean fluorescence intensity (MFI) of IL-1β. (f) Quantitative analysis of MFI of COX-2. (g) Quantitative analysis of MFI ratios of CD4 and CD8 (*n* = 3 per group, *P < 0.05, **P < 0.01, ***P < 0.001, n.s.: not statistically significant, one-way ANOVA).

To further elucidate the role of SPS in modulating wound inflammation, we analyzed the expression of pro-inflammatory cytokines in tissue samples collected on day 7 post-treatment. Immunofluorescence-based detection of inflammatory markers provided an indirect assessment of the extent of inflammation in the surrounding tissue. As shown in Fig. 4a–d, experimental groups treated with SPS in combination with either sunlight or 808 nm NIR light exhibited minimal expression of IL-1β, Foxp3, and IL-6 (Figs S19 and S20). In contrast, the control group displayed strong fluorescence signals, indicating pronounced inflammatory activity and suggesting that PDT treatment effectively mitigates inflammation (Fig. 4e). Furthermore, cyclooxygenase-2 (COX-2), a critical mediator of epidermal inflammation, was markedly upregulated in the control group, with expression levels elevated by ∼2–3-fold compared to the experimental group. Conversely, the experimental group exhibited a significant reduction in COX-2 expression, highlighting the potential anti-inflammatory and analgesic properties of PDT (Fig. 4f). Additionally, by comparing the fluorescence intensity of markers such as CD4/CD8 (Fig. 4g), a notable increase in epidermal positive signals was observed post-treatment. This may be linked to diabetes progression and could also be influenced by positive feedback from the inflammatory response [52]. CD4 cells function as critical immune sentinels, identifying potential threats and coordinating the mobilization of CD8 killer cells to target and eliminate pathogens. Elevated CD4 and CD8 levels also indicate the extent of pathogen-induced wounding [53]. In conclusion, these results suggest that SPS significantly enhances wound healing by modulating the inflammatory response.

M1 macrophages play a crucial role in type 1 helper T (Th1) cell recruitment, antimicrobial defense, and tumor suppression, primarily through innate and adaptive immune pathways. Their activation is predominantly triggered by pathogens, lipopolysaccharide (LPS), granulocyte-macrophage colony-stimulating factor (GM-CSF), tumor necrosis factor-alpha (TNF-α), and the Th1 cytokine interferon-gamma (IFN-γ) [54,55]. The absence of significant fluorescence signals for the surface antibody markers F4/80 and CD80, which are M1-type macrophage markers (Fig. S21), suggests that macrophages in the wound tissue did not undergo polarization to the M1 phenotype. This indicates that there was no excessive inflammatory response following treatment.

Evaluation of hemostasis and biosafety *in vivo.* The hemostatic efficacy of SPS was evaluated using a mouse tail amputation model (Fig. S22a). In the control group, continuous bleeding was observed, with a maximum blood loss of 98.2 mg, which was significantly reduced following SPS treatment. To further assess wound closure and the *in vivo* hemostatic potential of SPS, a mouse liver hemorrhage model was employed. In this model, blood loss in the SPS-PDT group was measured at 29.4 mg, ∼15% of that in the PBS control group (200.1 mg), indicating that SPS-PDT substantially enhances hemostasis *in vivo* (Fig. S22b–e). Histological analysis of liver tissue sections revealed that in the control group, the tissue surrounding the incision exhibited abnormal morphology with abundant free red blood cells, suggesting persistent hemorrhage. In contrast, SPS facilitated rapid hemostasis by promoting platelet aggregation, leading to the formation of a stable coagulation clot at the wound site. Additionally, clot formation effectively sealed the wound, preventing further internal bleeding. Transcriptome sequencing analysis of the thrombin signaling pathway further demonstrated that NF-κB upregulation is associated with platelet aggregation, coagulation, cell adhesion, and angiogenesis, potentially contributing to enhanced coagulation [56].

To assess the potential toxicity of SPS in different tissues, we collected heart, liver, spleen, lung, and kidney samples from different subgroups for H&E staining. No tissue damage was observed in either the SPS-treated or untreated groups (Fig. S23). Additionally, blood samples from SPS-treated mice were analyzed, and both routine blood tests and blood biochemistry parameters remained within the safe range (Figs S24 and S25). These findings demonstrate that SPS possesses good histocompatibility and biosafety while effectively facilitating hemostasis and wound healing.

### SPS produces analgesic effects

**Figure 5.**
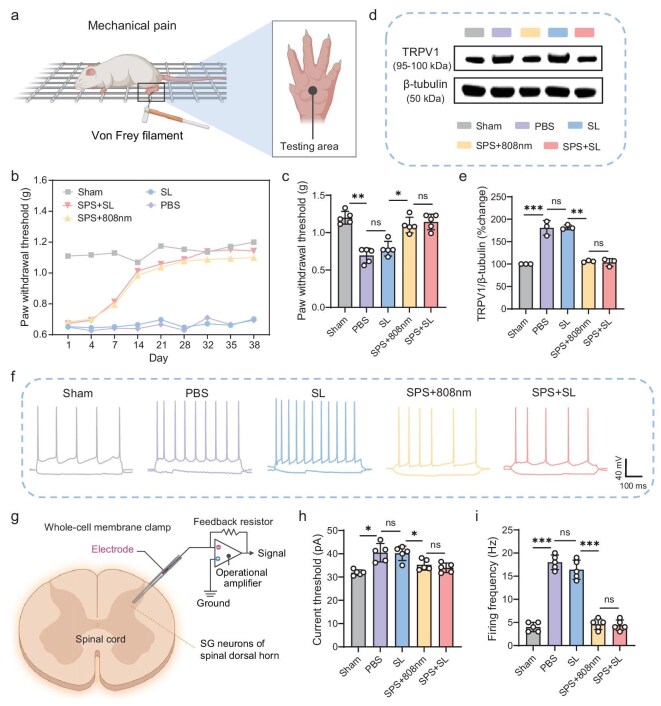
Analgesic effect of sunlight PDT. (a) Schematic representation of the mechanical pain test in mice. (b) Repeated measurements of mechanical sensitivity in the foot withdrawal response across different groups, tested every 3 days from day 1 to day 38 post-modeling, using nylon needles of 0.008, 0.4, 0.6, 1, 1.4, and 2 g. Each group included three replicates. Data represent changes in the withdrawals of the ipsilateral foot. (c) Statistical analysis of the number of paw withdrawals on the day 38. (d) Protein expression of TRPV1 in the lumbosacral spinal cord segment, with representative Western blotting bands of TRPV1 expression. (e) Statistical analysis of TRPV1/β-tubulin protein levels. Three samples were included in each group. (f) Representative traces of AP responses recorded from different experimental groups following a series of 500 ms hyperpolarizing and depolarizing current injections in 10 pA increments. Spinal cord tissue was collected 24 h post-treatment from each group for patch-clamp analysis (scale bar: 40 mV, 100 ms). (g) Schematic of the lumbar spinal cord slice, illustrating targeted recording of an SG neuron. The electrode represents a glass pipette targeting an SG neuron. (h) Statistical analysis of the current threshold in different groups. (i) Statistical analysis of firing frequency in different groups (*n* = 5 neurons from three mice per group, *P < 0.05, **P < 0.01, ***P < 0.001, n.s.: not statistically significant, one-way ANOVA).

To assess the analgesic effects of SPS, a plantar incision model was employed as a postoperative pain model, incorporating behavioral assessments alongside techniques such as Western blotting, membrane clamp analysis, and transcriptome sequencing. As illustrated in the experimental schematic, von Frey nylon filaments were used to measure mechanical pain at various time points [57] (Fig. 5a). Based on preliminary experiments, the model exhibited relatively consistent pain levels ∼1 day post-surgery. Consequently, pain behavior was assessed from the day 1 onwards to ensure stable, reliable, and comparable results. Treatment was initiated 30 h after the model was established, followed by subsequent treatments every 3 days. Mechanical pain thresholds were measured 24 h after each treatment by applying von Frey filaments of varying thickness, recording the pressure (g) at which a paw withdrawal response occurred, and calculating the pain threshold using an established formula. Two weeks after model establishment, the SPS-treated group, exposed to either sunlight or 808 nm light, exhibited a significant reduction in mechanical pain response compared to the groups treated with sunlight alone or PBS, with results approaching those of the sham group by week 5 (Fig. 5b and c).

In the pain signaling pathway, the dorsal root ganglion (DRG) and the dorsal horn of the spinal cord serve as pivotal regions for pain transmission [58]. Primary afferent neurons in the DRG detect noxious stimuli and relay pain signals to the dorsal horn, where they are subsequently propagated to higher centers in the nervous system, ultimately resulting in pain perception in the cortical areas. To investigate this process, we analyzed proteins from the L5 DRG 72 h post-incision surgery (66 h following drug administration) and the lumbosacral spinal cord segment. Western blotting was employed to quantify transient receptor potential vanilloid 1 (TRPV1), a critical mediator in the ascending transmission of pain through TRPV1 ion channels (Fig. 5d). Statistical analysis revealed a marked reduction in TRPV1 expression in the groups treated with sunlight or 808 nm light in combination with SPS, compared to those treated with sunlight or PBS alone. Notably, the group receiving SPS + sunlight exhibited the lowest TRPV1 expression, indicating that pain sensitivity in the mice returned to baseline following treatment with sunlight + SPS and 808 nm + SPS, with the most pronounced effect observed in the sunlight + SPS group (Fig. 5e).

Transient receptor potential (TRP) ion channels play a crucial role in sensory transduction and pain perception induced by injurious stimuli [52]. TRPV1 is a non-selective cation channel with high permeability to Ca^2+^. Therefore, its reduction may alter intracellular calcium signaling. Ca^2+^ can initiate neurotransmitter release at the synapse and modulate neuronal excitability. Neuronal excitability serves as a fundamental parameter of neuronal activity, reflecting a neuron’s capacity to generate action potentials (APs) [59]. Under painful conditions, the excitability of spinal substantia gelatinosa (SG) neurons is elevated, and their hyperexcitability plays a key role in the initiation and progression of pain. To assess this, whole-cell membrane clamp recordings were conducted to evaluate the excitability of spinal SG neurons across different experimental groups in the context of pain generation and transmission (Fig. 5f), allowing for direct, real-time, and quantitative measurement of charge movement of calcium ions through the channel [60]. Electrophysiological measurements were obtained from mice 72 h post-modelling. The threshold currents required to elicit APs and the frequency of APs evoked by a fixed depolarizing current were quantified across the groups. Notably, the frequency and total number of APs in sunlight PDT-treated mice were significantly lower compared to those in other treatment groups (Fig. 5g, h and i).

### SPS results in transcriptomic changes in bactericidal-, pain-, and coagulation-related pathways

To further analyze the role of SPS in the treatment model, we conducted transcriptome sequencing, focusing on the analysis and comparison of cellular factors associated with healing, pain, and hemostasis. The enriched Kyoto Encyclopedia of Genes and Genomes (KEGG) pathways are prominently highlighted (Fig. S26a, b and Figs S27–S29), revealing a comprehensive array of pathways related to these processes. Notably, we selected key genes implicated in various biological pathways, such as Th1 and Th2 cell differentiation, α-linolenic acid metabolism, ether lipid metabolism, cytosolic DNA-sensing pathways, extracellular matrix (ECM)-receptor interactions, phospholipase D signaling, cytokine-cytokine receptor interactions, calcium signaling, antigen processing and presentation, Fcγ receptor-mediated phagocytosis, Fc epsilon RI signaling, cell adhesion molecule (CAM)-PI3K-Akt signaling, natural killer cell-mediated cytotoxicity, B cell receptor signaling, complement and coagulation cascades, type I diabetes mellitus, NF-κB signaling, phagosome dynamics, and *Staphylococcus aureus* infection. The alignment and quality control of the sequenced genes are illustrated in Figs S30 and S31.

After PDT treatment, compared to the PBS control group, heatmap analysis (Fig. S26c) showed a significant decrease in pathways such as phospholipase D signaling, cytokine-cytokine receptor interaction, calcium signaling, antigen processing and presentation, B cell receptor signaling, complement and coagulation cascades, type I diabetes mellitus, and NF-γB signaling. In addition, relative mRNA expression was quantified using real-time quantitative polymerase chain reaction (qPCR) experiments in both treatment and control groups. As shown in Fig. S26d, NF-κB and IL-1β expression levels were significantly higher in the control group than in the treatment group. Further detection of immune factors is reflected in transcriptome sequencing data (Fig. S26e). These results suggest that SPS may promote wound healing and alleviate pain by reducing the inflammatory response, enhancing coagulation, and mitigating suffering.

## CONCLUSION

In conclusion, the SPS nanoparticle spray harnesses sunlight, an abundant and natural source for photodynamic therapy, achieving potent antibacterial activity and accelerated healing of diabetic wounds while effectively mitigating the risk of antibiotic resistance. SPS exhibits multifunctional capabilities, including rapid hemostasis, painless and efficient antibacterial effects (even against drug-resistant strains), modulation of inflammation, immune activation, and promotion of wound repair and analgesia. *In vitro*, MRSA and *E. coli* showed markedly reduced survival under sunlight exposure, with electron microscopy revealing severe structural damage to bacterial cells. *In vivo*, SPS combined with sunlight treatment reduced wound areas in diabetic mice to ∼20% of the PBS control, increased hair follicle density by ∼2.60-fold, and enhanced the collagen I/III ratio by ∼3.80-fold. Serum inflammatory markers TNF-α and IL-6 decreased to 14.0% and 45.6% of control levels, respectively, while wound tissue levels of IL-1β and COX-2 declined to 10.8% and 7.7%, and the CD4/CD8 ratio increased to 116.2% of the control value. Additionally, SPS demonstrated robust hemostatic effects, reducing tail and liver blood loss to ∼36.7% and ∼37.1% of control levels, shortening bleeding times, elevating mechanical pain thresholds by 1.65-fold, decreasing TRPV1 expression by 29.3%, and normalizing spinal neuron excitability. Transcriptomic and qPCR analysis further confirmed that SPS + sunlight therapy promotes wound healing and analgesia through modulation of inflammation, coagulation, and neural sensitivity.

Despite the inherent limitations of sunlight-mediated photodynamic therapy, including variability in intensity and exposure duration due to geographical, weather, and diurnal factors, as well as restricted tissue penetration, SPS nanoparticle spray provides a cost-effective, portable, and readily accessible therapeutic strategy, particularly suitable for emergency care and resource-limited settings. Moreover, the synthesis involves readily available and inexpensive starting materials, with a simple and straightforward self-assembly process that does not require complex equipment, indicating that scaling up is feasible. Overall, SPS demonstrates substantial clinical potential in diabetic wound management and offers a promising platform for the further development of photodynamic therapy and multifunctional nanomaterials.

## METHODS

Detailed experimental methods can be found in the online Supplementary file.

### Ethical statements

Adult male mice (20–28 g, Cyagen Biosciences) were housed in a temperature-controlled environment (26°C) with a 12-h light/dark cycle and *ad libitum* access to food and water. Diabetes was induced in 6-week-old mice via a high-sugar, high-fat diet (4–8 weeks), followed by intraperitoneal STZ injections (40 mg/mL, 5 days) in mice with fasting blood glucose ≥11 mmol/L. For the diabetic wound model, an 8 mm full-thickness excisional wound was created on the dorsal skin under isoflurane anesthesia. Sciatic nerve injury was induced by ligation of the sciatic nerve in the hind limb. All protocols were approved by the Animal Ethics Committee of Wuhan University (Approval No. WP20220020).

## Supplementary Material

nwaf554_Supplemental_File
