# Supplementary material for: Sunlight PDT leveraging NIR-II nanospray: painless, hemostatic, anti-inflammatory therapy towards diabetic wound infections
Source: Natl Sci Rev. 2025 Dec 5;13(2):nwaf554. doi: 10.1093/nsr/nwaf554 (PMC12839536; doi:10.1093/nsr/nwaf554)
Supplement: nwaf554_Supplemental_File [file nwaf554_supplemental_file.pdf]

Supporting Information

Sunlight PDT leveraging NIR-II nanospray: painless, hemostatic, anti-inflammatory therapy towards diabetic wound infections

Qihang Ding[1,†], Lingbo Zhou[2,4,†], Tao Xiong[3,†], Jiqiang Liu[5,†], Luojia Chen[2], Jiyoung Yoo[1], Xiaoyu Xu[2], Xianglei Jia[4], Siling Chen[2], Siyu Chen[2], Yifan Wang[6], Ping Gong[5,*], Meijia Gu[2,*], Wen Jiang[5,*] and Jong Seung Kim[1,*]

[1]Department of Chemistry, Korea University, Seoul 02841, Republic of Korea;

[2]Brain Center, Department of Neurosurgery, Ministry of Education Key Laboratory of Combinatorial Biosynthesis and Drug Discovery, Zhongnan Hospital of Wuhan University, School of Pharmaceutical Sciences, Wuhan University, Wuhan 430071, China;

[3]College of Chemistry and Chemical Engineering, Central South University, Changsha 410083, China;

[4]Renmin Hospital of Wuhan University, Wuhan 430060, China;

[5]Guangdong Key Laboratory of Nanomedicine, Chinese Academy of Sciences-Hong Kong Joint Lab for Biomaterials, Chinese Academy of Sciences Key Laboratory of Biomedical Imaging Science and System, Center for Nanomedicine and Nanobiotechnology, Institute of Biomedicine and Biotechnology, Shenzhen Institutes of Advanced Technology, Chinese Academy of Sciences, Shenzhen 518055, China;

[6]Department of Radiation Oncology, The University of Texas MD Anderson Cancer Center, Houston, TX 77030, USA

[+] These authors contribute equally to this work.

* Corresponding authors:

Prof. Ping Gong, Email: ping.gong@siat.ac.cn

Prof. Meijia Gu, Email: mjgu@whu.edu.cn

Prof. Wen Jiang, Email: WJiang4@MDAnderson.org

Prof. Jong Seung Kim, E-mail: jongskim@korea.ac.kr

1. Materials and methods

1.1. Materials and Characterization

All chemical reagents utilized in this study were procured from reputable commercial suppliers, including Aldrich, TCI, Adamas, and Energy Chemical, and were employed without further purification unless otherwise specified. Spirulina platensis was obtained from Guangyu Biological Technology (Shanghai, China). Confocal fluorescence imaging was conducted using a laser scanning microscope equipped with a two-photon *in vivo* imaging system and a Leica SPE platform. UV-Vis-NIR absorption spectra were recorded using a Shimadzu UV-3600 spectrophotometer. TEM images were acquired on an HT7700 TEM at an acceleration voltage of 100 kV. NIR-II fluorescence spectra were measured using a Fluorolog 3 spectrofluorometer (Horiba) with an 808 nm diode laser. NIR-II fluorescence imaging was performed using a dedicated imaging system (Wuhan Grand Imaging Technology Co., Ltd.). NMR spectra were recorded on a Bruker 400 MHz spectrometer for ^{1}H NMR and at 100 MHz for ^{13}C NMR, with chemical shifts referenced to tetramethylsilane (TMS) as an internal standard. Electrospray ionization mass spectrometry (ESI-MS) spectra were obtained using a Thermo Fisher Scientific Finnigan LCQ Advantage system. EPR spectra were measured using a Bruker ELEXSYS-II E500 CW-EPR spectrometer.

1.2. Photostability Test of QH and SPS

The photostability of QH and SPS was evaluated by monitoring fluorescence intensity over time following continuous laser irradiation. In the experiment, a small quantity of QH and SPS was dissolved in DCM and placed in a centrifuge tube. The samples were subjected to irradiation using an 808 nm laser at a power density of approximately 1.0 W/cm^2, and fluorescence intensity was recorded at designated time points (0, 5, 10, 15, 20, 25, and 30 min). For comparison, the photostability of ICG was assessed under identical conditions as a control.

1.3. Detection of ROS generation

The levels of ROS, •OH, and $O_2^{\bullet -}$ were assessed using DCFH-DA, HPF, and DHR123 as respective indicators. A near-infrared 808 nm laser (1.0 W/cm^2) or sunlight irradiation (45.5 mW/cm^2) was applied to 1 mL of PBS containing activated DCFH-DA (20 μM), HPF (20 μM), or DHR123 (20 μM), along with QH, SPS, or ICG (20 μM). Fluorescence spectra of DCFH-DA, HPF, and DHR123 were recorded at an excitation wavelength of 488 nm, with emission intensities measured at 525 nm.

1.4. In situ EPR Spectrum of QH and SPS

EPR spectroscopy was employed to detect radical species in PBS using the spin-trapping agent DMPO. In the experiment, DMPO was combined with QH and SPS at a concentration of 20 μM and subsequently exposed to sunlight irradiation (~45.5 mW/cm^2) for 5 minutes. The resulting EPR spectra were then recorded to analyze radical formation.

1.5. Theoretical calculations

Theoretical calculations were conducted using the Gaussian 09 package with molecular visualization facilitated by GaussView. The frontier molecular orbitals (HOMO/LUMO) and corresponding energy levels of the investigated compounds were computed using TD-DFT at the B3LYP/6-31G(d) level of theory.

Additionally, SOC values were evaluated employing TD-DFT at the B3LYP/def2-TZVP level, as implemented in ORCA 5.0.

1.6. Bacterial killing experiment

Bacterial suspensions with a concentration of 10^7 CFU/mL were prepared, with 0.5 mL of the bacterial solution mixed with 0.1 mL of dye buffer (comprising stock solution and buffer). The mixtures were incubated at 37°C for 30 min under constant shaking. Subsequently, samples were exposed to simulated sunlight at an intensity of 50 mW/cm^2 for 10 min (corresponding to natural sunlight conditions around 3:00 p.m., Beijing time). Control groups included a PBS-treated group and a dark group, both subjected to identical incubation conditions without light exposure. Post-irradiation, the samples were plated on agar and incubated at 37°C overnight to assess bacterial viability. The strains tested included E. coli (ATCC 8739) and MRSA

1.7. SYTO9-PI Live and Dead Bacteria Stain Kit

The dye mixture was prepared by combining SYTO9 and PI solutions in a 1:1 volume ratio within a centrifuge tube. For control samples, 1 mL of live bacterial suspension and 1 mL of heat-killed (dead) bacterial suspension were each mixed with 3 μL of the dye mixture. Test samples were similarly prepared by suspending bacteria in 1 mL of 0.85% NaCl solution, followed by the addition of 3 μL of the dye mixture. All samples were incubated in the dark at room temperature for 15 min to prevent photobleaching. Subsequently, 5 μL of each stained suspension was placed onto a glass slide, covered with an 18 mm square coverslip, and examined using fluorescence microscopy. Live bacteria exhibited green fluorescence under a fluorescein filter, while dead bacteria displayed red fluorescence under a Texas Red filter.

1.8. E. coli motility analysis

E. coli was subjected to six distinct treatments: PBS, PBS with sunlight exposure, PBS with 808 nm laser irradiation, SPS in the dark, SPS with sunlight, and SPS with 808 nm irradiation, each for a duration of 20 min post-treatment, the bacteria were fluorescently labeled using NucGreen, with excess dye removed via PBS washing. The labeled bacteria were then transferred to confocal dishes for immediate imaging using time-lapse live confocal microscopy (11 frames captured at 2-s intervals over 20 s). The motility of multidrug-resistant Pseudomonas aeruginosa (MDR-PA) was simultaneously recorded. Bacterial adhesion and motility were quantified through video analysis using the tracking plug-in of NIS-Elements software. Individual bacterial trajectories were tracked to determine displacement distances and extract motility-related parameters. MSD values were directly obtained from NIS-Elements, and the corresponding α values were subsequently calculated.

$$\alpha = \frac{logMSD}{log\Delta t}$$

1.9. Animal and Disease Models

Adult male mice (20-28 g, Cyagen Biosciences) were housed in a temperature-controlled environment (26°C) with a 12-hour light/dark cycle and ad libitum access to food and water. All protocols were approved by the Animal Ethics Committee of Wuhan University (Approval No. WP20220020). Diabetes was induced

in six-week-old mice via a high-sugar, high-fat diet (4–8 weeks), followed by intraperitoneal STZ injections (40 mg/mL, 5 days) in mice with fasting blood glucose ≥ 11 mmol/L. For the diabetic wound model, an 8-mm full-thickness excisional wound was created on the dorsal skin under isoflurane anesthesia. Sciatic nerve injury was induced by ligation of the sciatic nerve in the hind limb.

1.10. Animal behavior test

For the von Frey test, mice were acclimated in individual boxes on a metal mesh floor for two days before baseline measurements. On the testing day, the mice underwent a 30-minute habituation period. Von Frey filaments (0.008, 0.4, 0.6, 1, 1.4, 2 g) were applied perpendicularly to the hind paw plantar surface near the incision site, with five stimulations per filament (1 s each, 1 min intervals). The minimal force eliciting a withdrawal response was recorded as the mechanical threshold, and the force preceding this response as the mechanical pain threshold. The 50% PWT was calculated using Dixon's up-and-down method. All assessments were performed by blinded experimenters.

1.11. SEM

Bacterial samples were washed three times with PBS and fixed in 2.5% glutaraldehyde (4°C, overnight). Following post-fixation washes with PBS, samples underwent dehydration through graded ethanol (30%, 50%, 75%, 90%, 95%, 100%) and increasing concentrations of tert-butanol (30%, 50%, 70%, 90%, 100%). After complete dehydration, samples were dried and sputter-coated for conductivity. SEM imaging was conducted using a Zeiss Sigma 300 microscope to analyze bacterial morphology.

1.12 H&E staining

Mice were euthanized, and tissues or organs were harvested for histological evaluation. The collected specimens were fixed in a suitable fixative at 4°C overnight, followed by dehydration through a graded ethanol series (80%, 90%, 95%, and 100%). The dehydrated tissues were embedded in paraffin and sectioned into 4 μm slices. Tissue sections underwent deparaffinization, rehydration, and subsequent H&E staining. The stained slides were examined under a Nikon E100 microscope to assess histological alterations across experimental groups.

1.13. Immunofluorescence staining

At the end of the six-day period, the mice were euthanized, and wound tissues were harvested for histological analysis. Tissues were fixed in ocular fixative (overnight, 4°C), dehydrated through graded ethanol (80%, 90%, 95%, 100%), embedded in paraffin, and sectioned into 4-μm slices. Sections were permeabilized (0.3% Triton X-100 in PBS, 15 min) and blocked with 5% sheep serum (1 h, RT). Primary antibodies against CD4, CD8, IL-1β, IL6, COX2, and Foxp3 were applied overnight (4°C), followed by Alexa Fluor-conjugated secondary antibodies (1:400, 1 h, RT). Nuclei were stained with DAPI (1:500). Fluorescent images were captured, and expression intensities were quantified using ImageJ.

1.14. Sirius red staining

The levels of two collagen types were assessed using Sirius red staining. The prepared tissue sections were incubated with Sirius red reagent for 30 minutes at room temperature. Following incubation, the color was eluted using 1 mL of a 1:1 mixture of NaOH and absolute methanol. Collagen content was quantified via

polarized light microscopy, and analysis was conducted using ImageJ software. Type I collagen appeared orange-yellow or light red under polarized light, while type III collagen was stained green.

1.15. Western blot

Spinal cords were harvested from euthanized mice, snap-frozen at −80°C, and homogenized in an ice-cold RIPA buffer with protease/phosphatase inhibitors (KZ-5F-3D, Servicebio). Lysates were centrifuged (13,000 × g, 10 min, 4°C), and supernatants were collected for protein quantification via BCA assay. Equal protein amounts were denatured (100°C, 10 min), separated by SDS–PAGE (10% gels), and transferred to PVDF membranes. After blocking with 5% non-fat milk in TBST (1 h, RT), membranes were incubated with primary antibodies against TRPV1 and β-Tubulin (4°C, overnight), followed by HRP-conjugated secondary antibodies (1 h, RT). Bands were detected using ECL and imaged with a ChemiDoc™ XRS+ system (Bio-Rad).

1.16. In vivo biosafety assessment

SPS (50 μg/g body weight) was administered via tail vein injection in mice (n=3). After 28 days, the mice were euthanized, and blood samples were collected for routine hematological analysis. Concurrently, major organs—including the heart, liver, spleen, lungs, kidneys, and brain—were harvested for histopathological evaluation using H&E staining.

1.17. Evaluation of hemostasis

The *in vivo* hemostatic performance of SPS was evaluated using Celox as a commercial comparator. Mice were anesthetized and secured in a supine position, followed by a midline laparotomy to expose the median lobe of the liver. Pre-weighed filter paper was placed beneath the liver to facilitate quantification of blood loss. A uniform 6 mm incision was made along the liver midline using a sterile scalpel. Immediately post-injury, 50 μg/mL SPS was applied directly to the bleeding site. Animals receiving no treatment served as negative controls. Blood loss and time to achieve hemostasis were recorded throughout the procedure. For histopathological assessment, liver tissues from five experimental groups were collected and processed for H&E staining following standard protocols. Briefly, liver samples were fixed in 4% formaldehyde for 24 h, dehydrated through a graded ethanol series (70%, 80%, 95%, and 100%), cleared in xylene, and embedded in paraffin. Sections of 5 μm thickness were prepared, deparaffinized in xylene, and rehydrated through descending ethanol concentrations. The sections were subsequently stained with hematoxylin and eosin and examined using an optical microscope (Olympus BX53). Additionally, the hemostatic efficacy of SPS was assessed in a murine tail amputation model. Under anesthesia, 30% of the proximal tail length was transected, and the stump was exposed to ambient air for 5 s prior to SPS application. Hemostatic performance was determined by measuring blood loss within 90 s post-amputation.

1.18. Evaluation of wound healing effects

Mice were randomly assigned into five groups with equal numbers. All surgical procedures were conducted under anesthesia. Following dorsal hair removal, two symmetrical full-thickness excisional wounds (10 mm in diameter) were created on the back of each mouse. SPS (50 μg/mL) was uniformly applied to the wound surface, while control groups received treatments with PBS, and vancomycin (300 μg/mL). Wound healing progression was monitored and documented through serial photographic imaging

from day 0 to day 14. At designated time points, wound bed tissues were harvested, fixed in formaldehyde, and processed for histological examination via H&E staining. Wound area measurements were performed using ImageJ software, and wound closure rates were calculated accordingly. Hair follicle regeneration was assessed by counting follicles in three independent H&E-stained sections per group at 4× magnification. The width of immature granulation tissue was quantified using ImageJ, with values averaged across three separate sections per group. Epidermal thickness was measured at three distinct sites per sample using ImageJ on three independent H&E-stained sections per group. Additionally, neovascularization and inflammatory responses were analyzed via immunofluorescence staining. Quantitative fluorescence intensity was assessed using ImageJ on three representative images per group, with mean intensity values calculated within defined regions of interest (ROIs) surrounding the wound area at 20× magnification. Fluorescence intensity values were normalized to the corresponding wound area for each ROI.

1.19. Spinal cord section with whole-cell membrane clamp recording

Adult mice were anesthetized with sodium pentobarbital and euthanized via cervical dislocation. Lumbar spinal cord segments (L4–L5) were rapidly isolated and immersed in ice-cold, oxygenated artificial cerebrospinal fluid (ACSF; 95% O_2/5 % CO_2) containing (in mM): 92 NMDG, 2.5 KCl, 1.25 NaH_2PO_4, 30 $NaHCO_3$, 20 HEPES, 25 glucose, 2 thiourea, 5 sodium ascorbate, 3 sodium pyruvate, 0.5 $CaCl_2 \cdot 2H_2O$, and 10 $MgSO_4 \cdot 7H_2O$ (pH 7.3, 295–305 mOsm). Transverse slices (300 μm) were prepared using a vibratome (VT1200S, Leica Microsystems) and incubated at 33°C for 10 min in oxygenated NMDG-ACSF, then maintained at room temperature in HEPES-buffered ACSF for ≥1 h. Recordings were performed in slices perfused with oxygenated ACSF (in mM: 125 NaCl, 2.5 KCl, 2 $CaCl_2$, 1 $MgCl_2$, 1.25 NaH_2PO_4, 25 $NaHCO_3$, 12.5 D-glucose; pH 7.3–7.4) at 2 mL/min.

1.20. Whole-cell patch-clamp recording

Whole-cell patch-clamp recordings were conducted on spinal dorsal horn SG neurons using differential interference contrast optics (BX51WI, Olympus, Japan) in current- and voltage-clamp modes. Recording pipettes (6–8 MΩ) were pulled from borosilicate capillaries (P-1000, Sutter Instruments) and filled with internal solution containing (mM): 120 K-gluconate, 5 NaCl, 10 KCl, 1 $CaCl_2$, 2 $MgCl_2$, 11 EGTA, 10 HEPES, 2 Mg-ATP, and 1 Li-GTP (pH 7.3, adjusted with Tris-base). After achieving whole-cell configuration, neurons equilibrated for 5 min. Resting membrane potential was monitored in current-clamp mode (I = 0). Action potential (AP) thresholds were determined via 500 ms current injections (−40 to 100 pA, 10 pA steps), with the threshold defined as the minimal current evoking an AP. APs were also recorded in response to 100 pA depolarizing ramps (1 s). For sEPSC recordings, cells were voltage-clamped at −70 mV. Signals were amplified (MultiClamp 700B, Molecular Devices), filtered at 2 kHz, and digitized at 10 kHz (pClamp 10.7, Molecular Devices). Only neurons with access resistance < 20 MΩ and input resistance > 100 MΩ were analyzed; cells with >20% fluctuation in these parameters were excluded.

1.21. Transcriptome sequencing

Adult mice were anesthetized with sodium pentobarbital and euthanized via cervical dislocation. Lumbar spinal cord segments (L4–L5) were rapidly isolated and immersed in ice-cold, oxygenated artificial cerebrospinal fluid, then extracting RNA and testing for purity (NanoPhotometer spectrophotometer) and

integrity (Agilent 2100 bioanalyzer). The total amount of RNA after library construction was less than 1μg (NEBNext® UltraTM RNA Library Prep Kit). After the library inspection was qualified (Agilent 2100 bioanalyzer), different libraries were pooled according to the requirements of the effective concentration and the targeted amount of data to be obtained from the sequencing machine, and then Illumina sequencing was performed. The image data of the sequencing fragments measured by the high-throughput sequencer were converted into sequence data (reads) through base calling by CASAVA, and the file was in FASTQ. Format.

1.22. Statistical methods

All experiments were conducted with a minimum of three independent replicates. Data were analyzed using GraphPad Prism 8 statistical software. Two-dimensional images and fluorescence intensities were processed and analyzed with ImageJ. Three-dimensional images were processed using NIS-Elements AR software (Nikon) and COMSTAT 2.0. Data are presented as means ± SD unless stated otherwise. Statistical significance was assessed using an unpaired t-test or ANOVA in GraphPad Prism 8, with significance levels indicated as follows: $p < 0.05$ (*), $p < 0.01$ (**), $p < 0.001$ (***), $p < 0.0001$ (****), and n.s.=not significant.

Figures

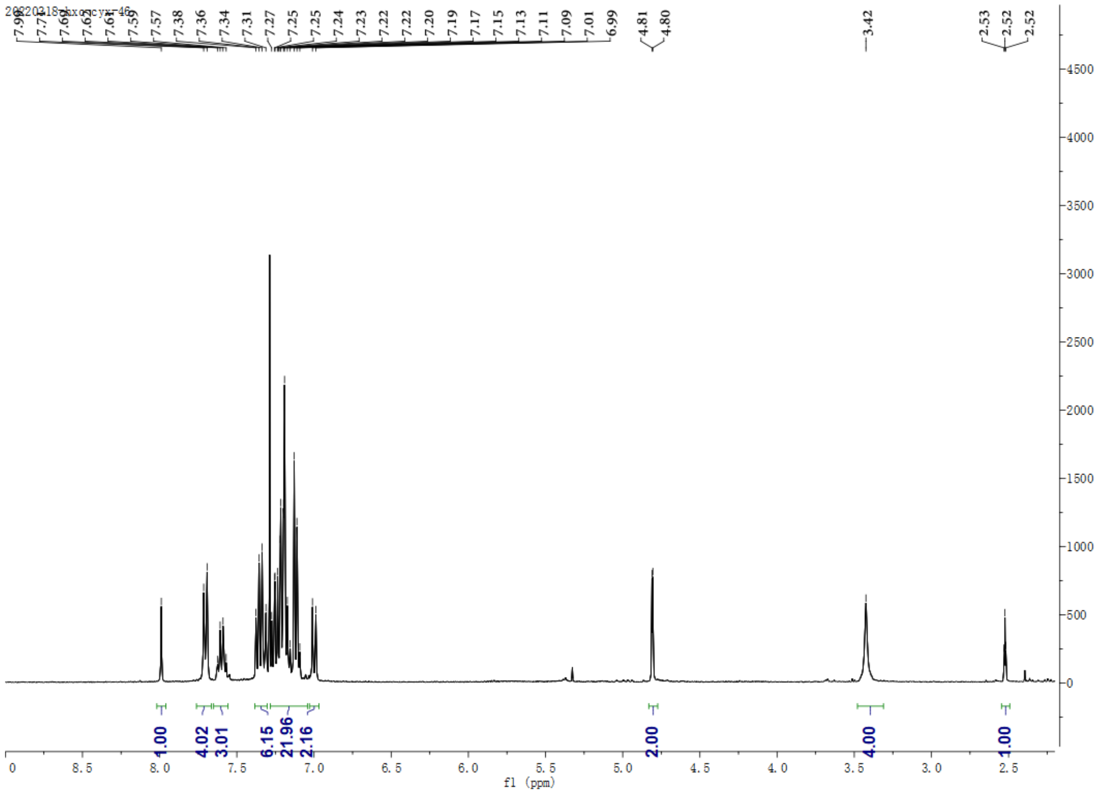

Fig. S1 ^{1}H NMR Spectrum of **QH** in Acetonitrile-d_3.

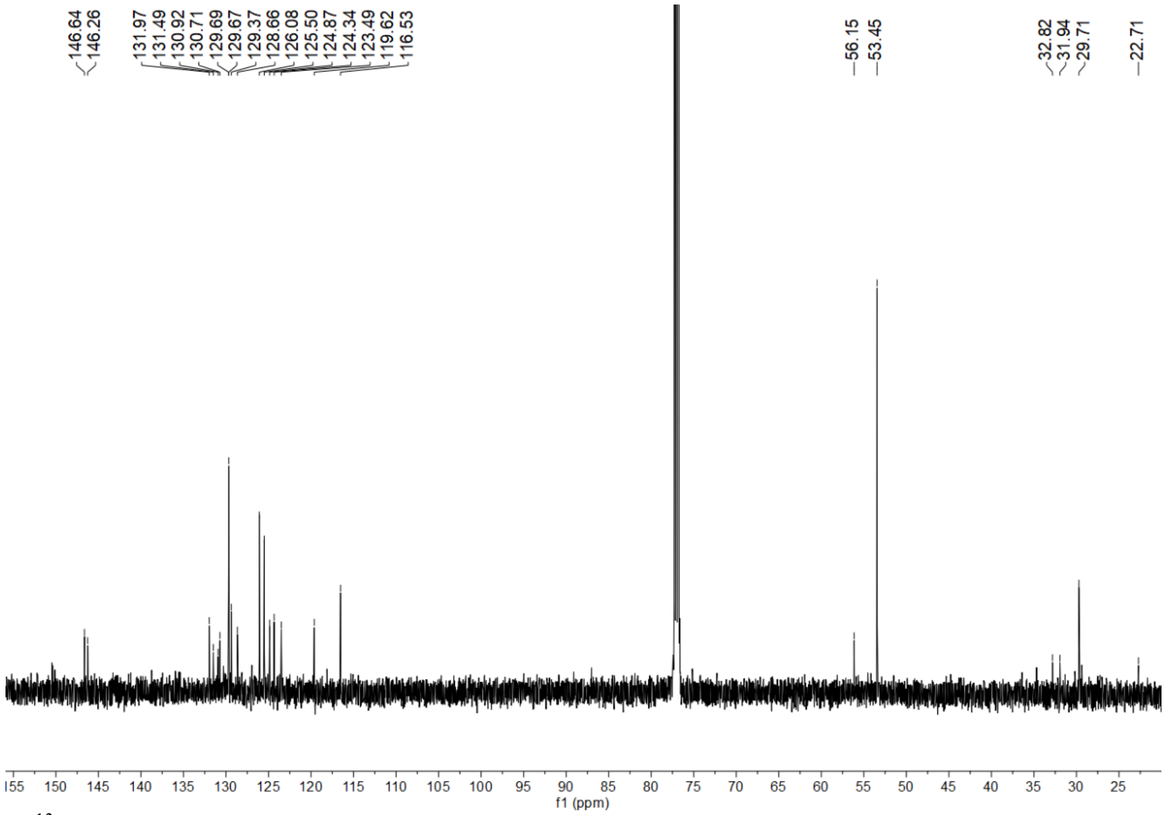

Fig. S2 ^{13}C NMR Spectrum of **QH** in Chloroform-*d*.

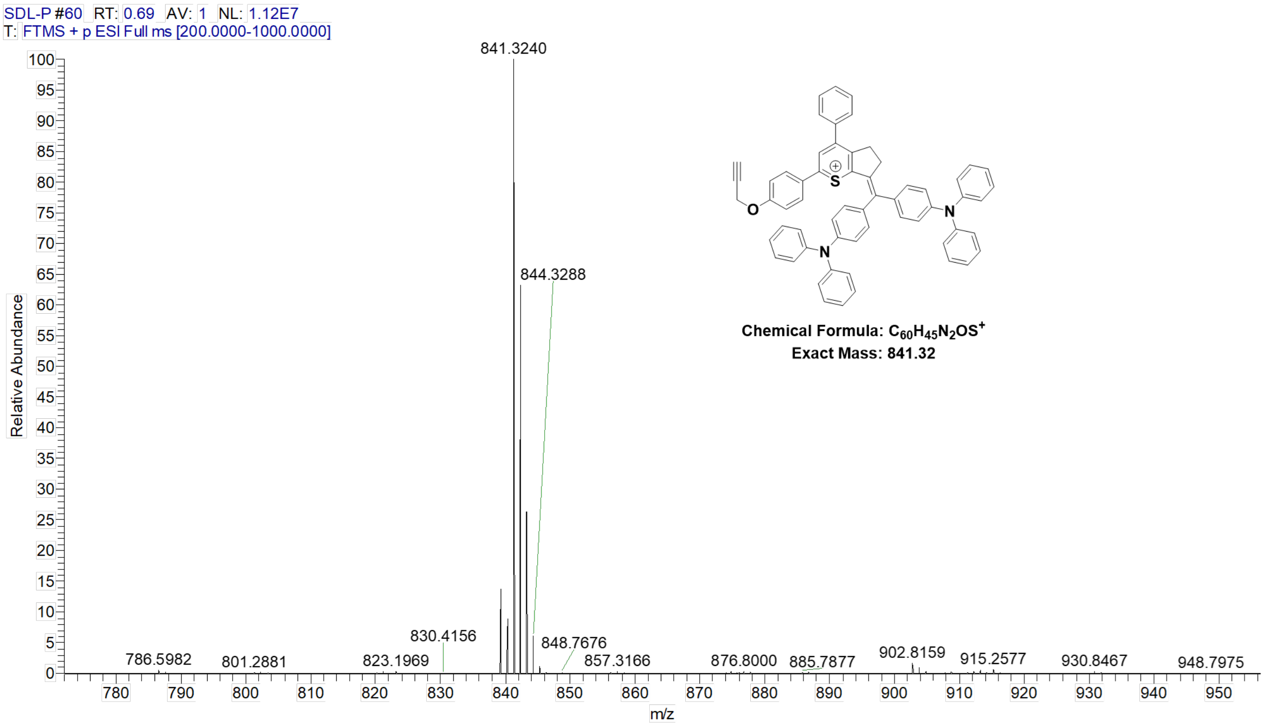

Fig. S3 ESI-MS spectrum of **QH**.

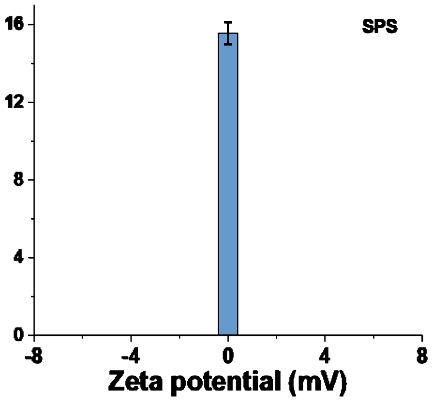

Fig. S4 Zeta potential of **SPS** (*n*=3).

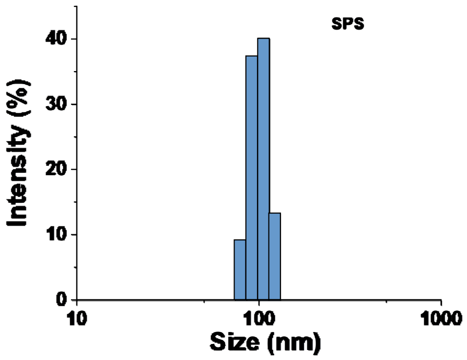

Fig. S5 DLS size distribution after irradiation by 30 min 808 nm laser (1W/cm^2)

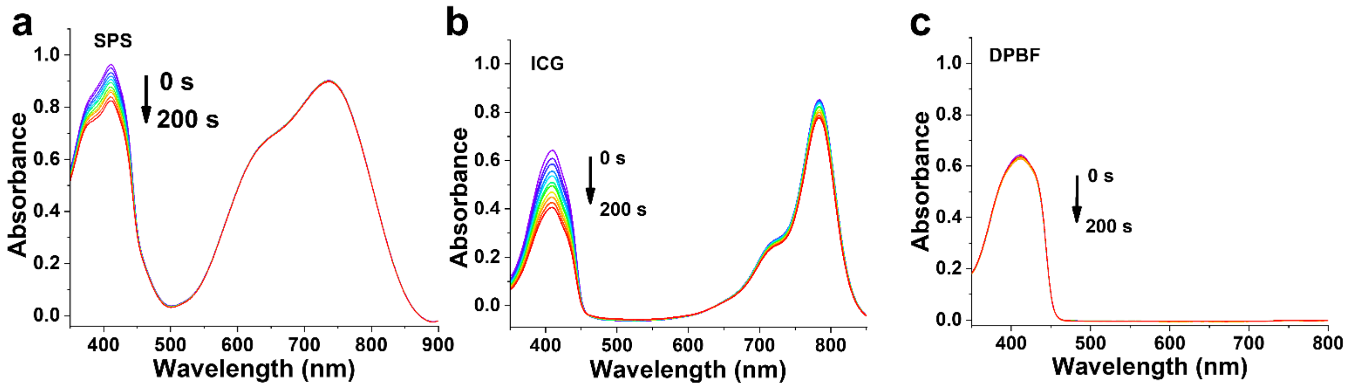

Fig. S6 Singlet oxygen assay using the decreased absorbance of DPBF at 410 nm in the presence of (a) **SPS** (20 µM), (b) ICG (20 µM), (c) DPBF in MeOH at room temperature under 808 nm laser excitation (1 W/cm^2).

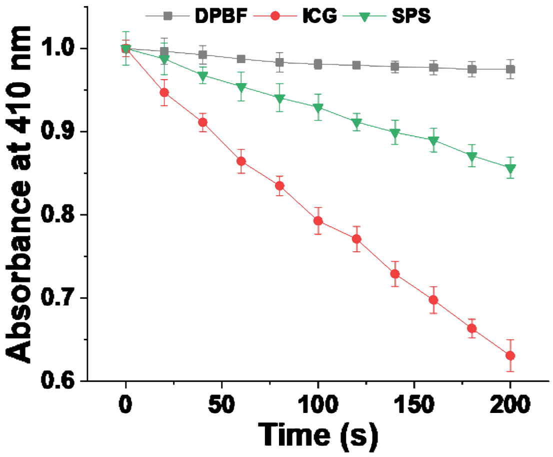

Fig. S7 Absorbance change of DPBF at 410 nm with time in the presence of **SPS** and ICG, respectively, in MeOH at room temperature under 808 nm laser excitation (1 W/cm^2).

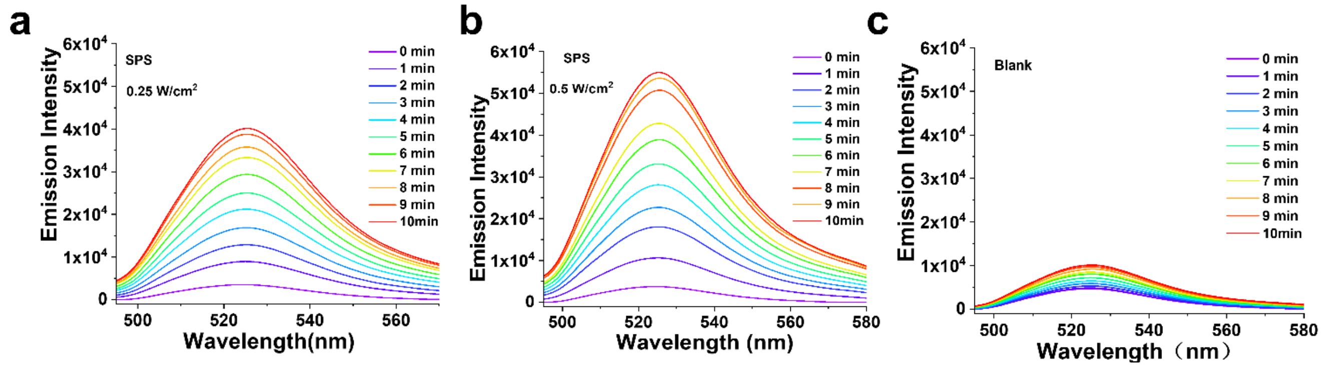

Fig. S8 Emission spectra of DHR123 at different irradiated times in the presence of **SPS** (20 μM) with different power ((a) 0.25 (b) and 0.5 W/cm^2), respectively, and (c) blank DHR123 (0.5 W/cm^2) under 808 nm laser excitation.

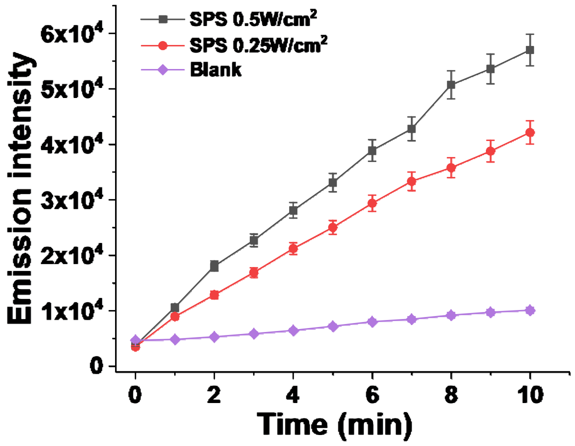

Fig. S9 Emission intensity of DHR123 at 525 nm at different irradiation times of **SPS** in aqueous solution under 808 nm laser with different power intensity (0.25 and 0.5 W/cm^2), respectively (the concentration of **SPS** is 20 μM).

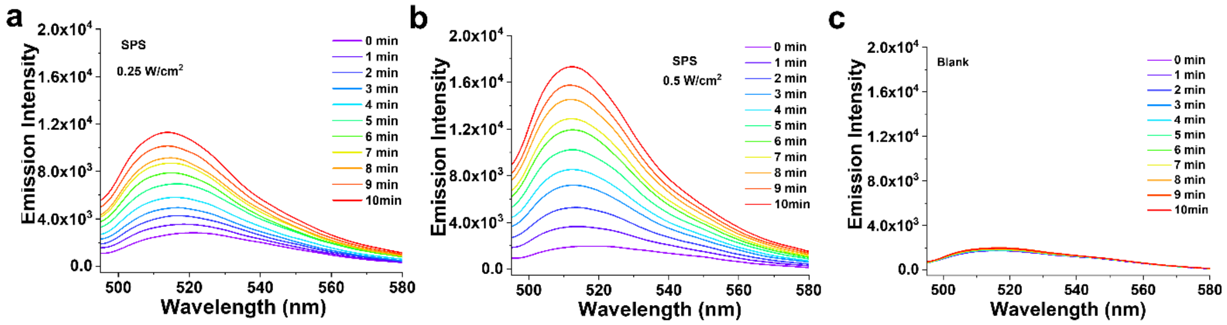

Fig. S10 Emission spectra of HPF at different irradiated times in the presence of **SPS** under 808 nm laser excitation with different power ((a) 0.25 and (b) 0.5 W/cm^2), respectively, and (c) blank DHR123 (0.5 W/cm^2). (The concentration of **SPS** is 20 μM).

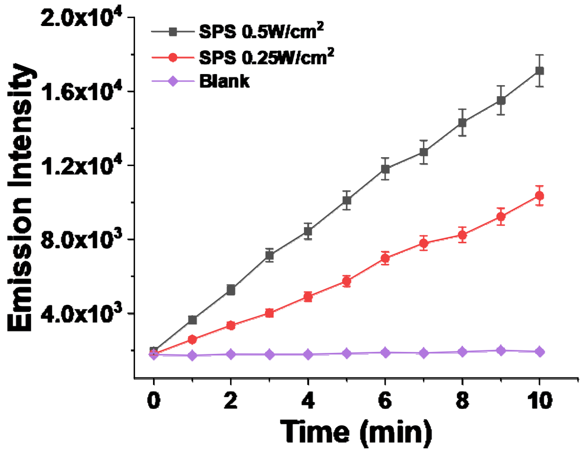

Fig. S11 Emission intensity of HPF at 525 nm at different irradiation times of **SPS** in aqueous solution under 808 nm laser with different power intensities (0.25 and 0.5 W/cm^2), respectively. (The concentration of **SPS** is 20 μM).

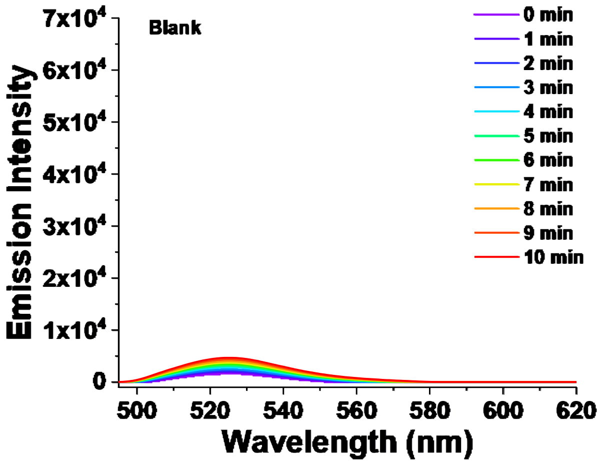

Fig. S12 Emission intensity of DFR123 at different irradiation times under sunlight (45.5 mW/cm^2).

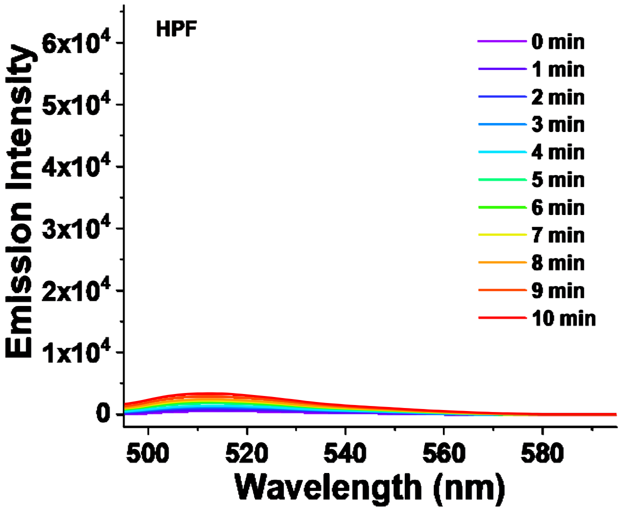

Fig. S13 Emission intensity of HPF at different irradiation times under sunlight (45.5 mW/cm^2).

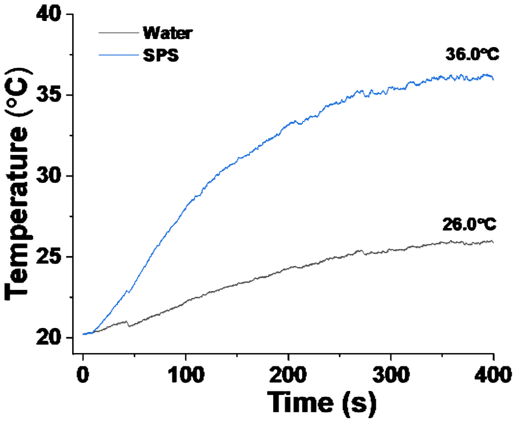

Fig. S14 Temperature change curves of **SPS** (100 μM) under the laser irradiation (808 nm, 1 W/cm^2).

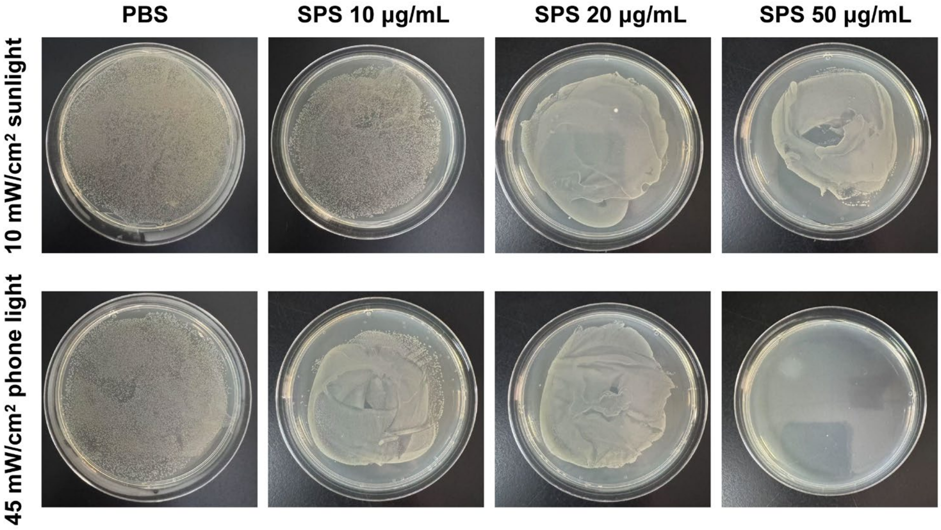

Fig. S15 The evaluation of SPS antibacterial effects against *E. coli* under conditions of insufficient light on cloudy days (10 mW/cm^2) and an alternative smartphone lighting setup indoors (aroud 45 mW/cm^2).

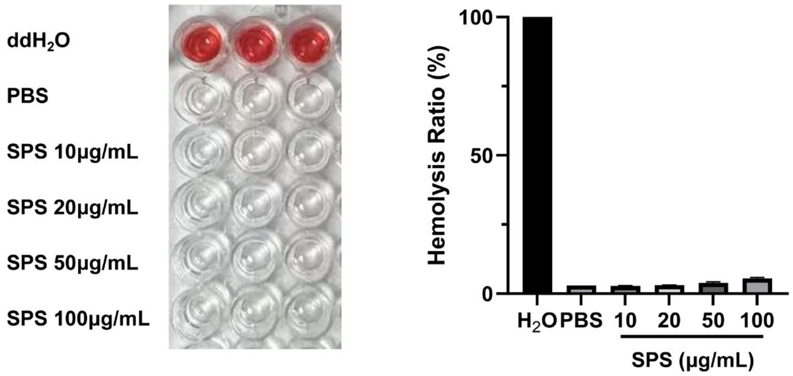

Fig. S16 Hemolysis experiments for blood of mice treated with SPS of 10 μg/mL, 20 μg/mL, 50 μg/mL, 100 μg/mL, compared with ddH_2O and PBS.

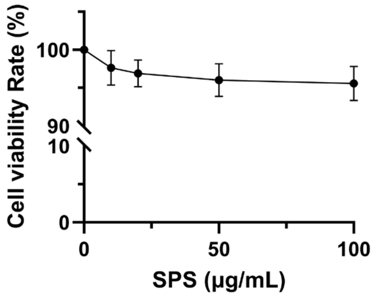

Fig. S17 CCK8 assay for HEK293 cell treated with SPS of 10 μg/mL, 20 μg/mL, 50 μg/mL, 100 μg/mL, compared with PBS.

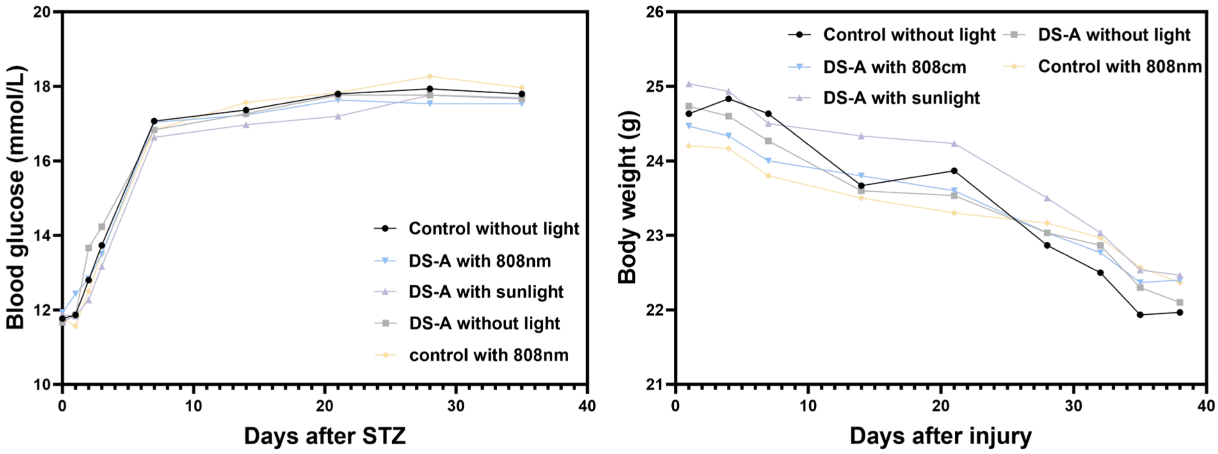

Fig. S18 a) Blood glucose of mice from 5 groups. b) Body weight of mice from 5 groups (n = 3 each group).

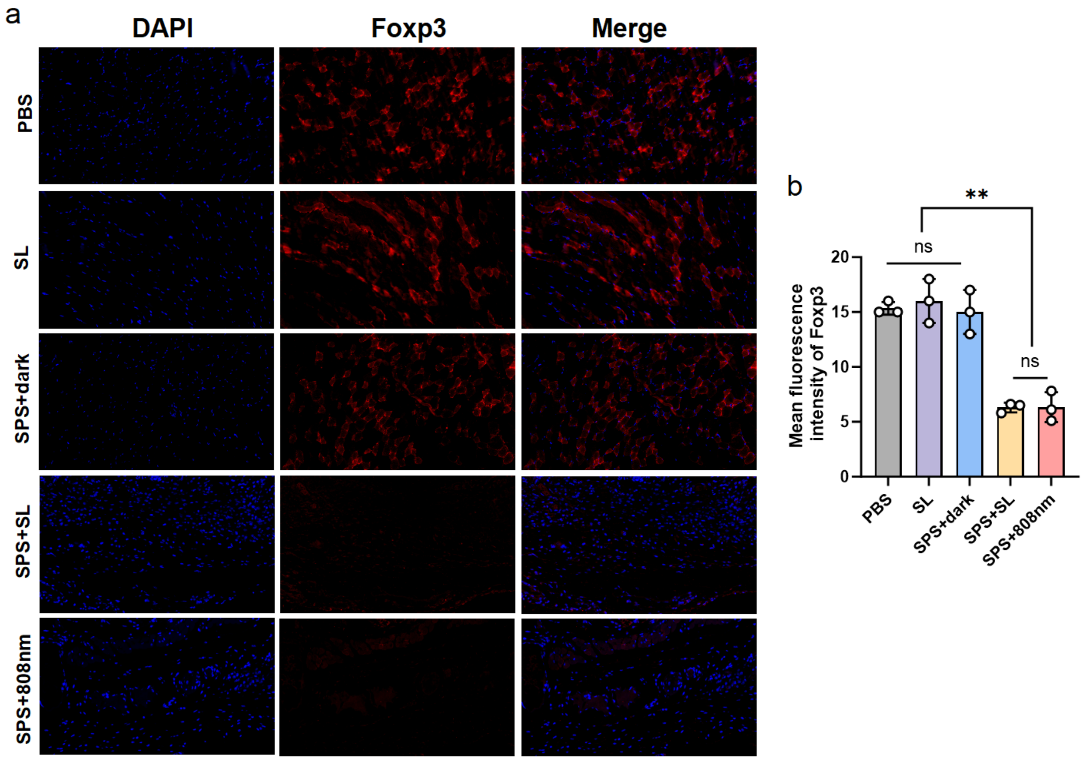

Fig. S19 (a) Representative immunofluorescence images of Foxp3 in skin tissue at different subgroups. Co-labeling of DAPI. (b) Quantitative statistics of fluorescence of Foxp3 (n = 3, *p < 0.05, **p < 0.01, ***p < 0.001, n.s.: no statistically significant, one-way ANOVA).

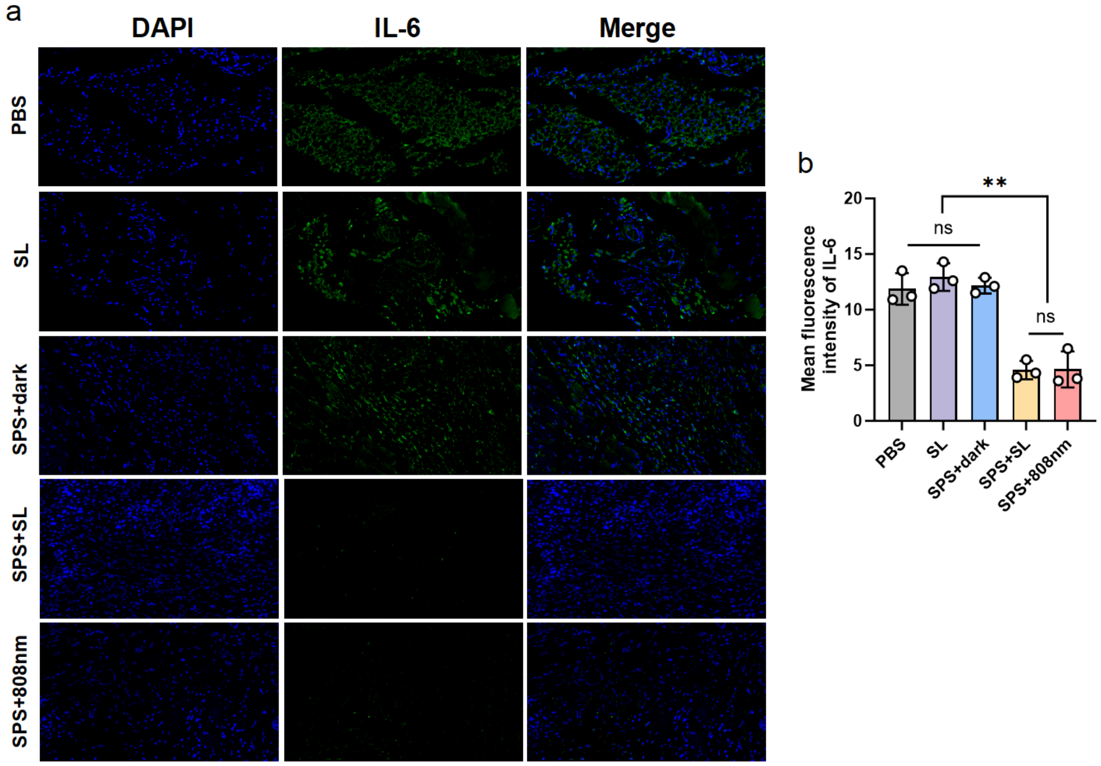

Fig. S20 (a) Representative immunofluorescence images of IL-6 in skin tissue at different subgroups. Co-labeling of DAPI. (b) Quantitative statistics of fluorescence of IL-6 (n = 3, *p < 0.05, **p < 0.01, ***p < 0.001, n.s.: no statistically significant, one-way ANOVA).

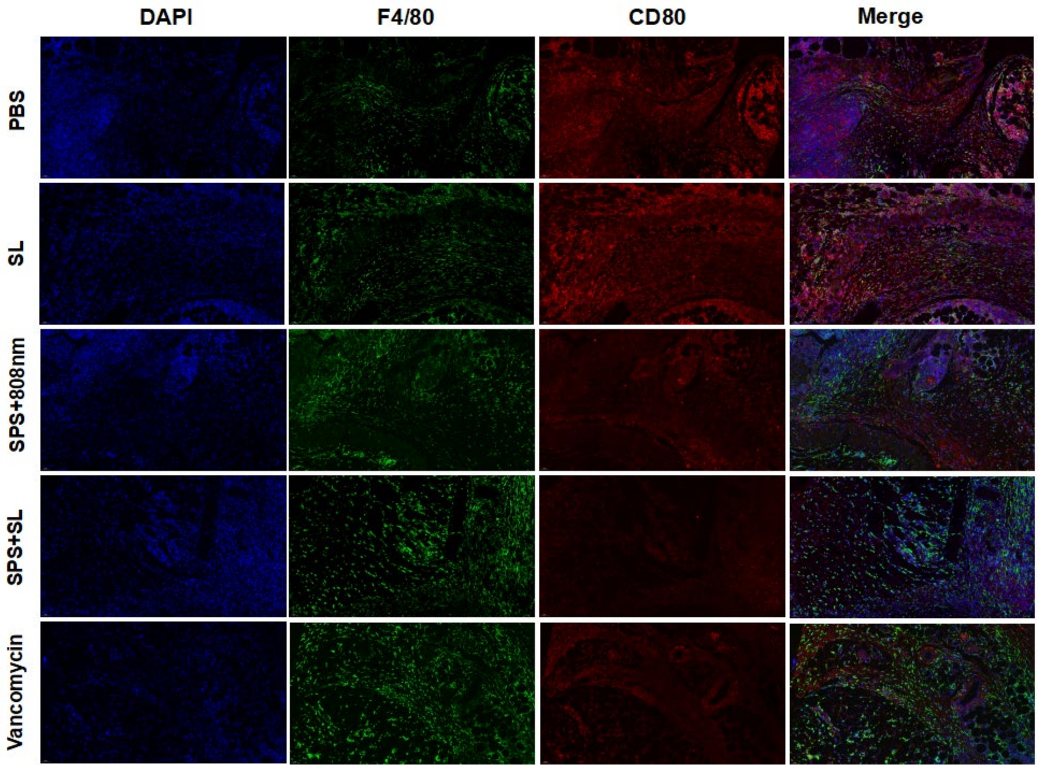

Fig. S21 Immunofluorescence images of F4/80 and CD80 in skin tissue at different subgroups. Co-labeling of DAPI.

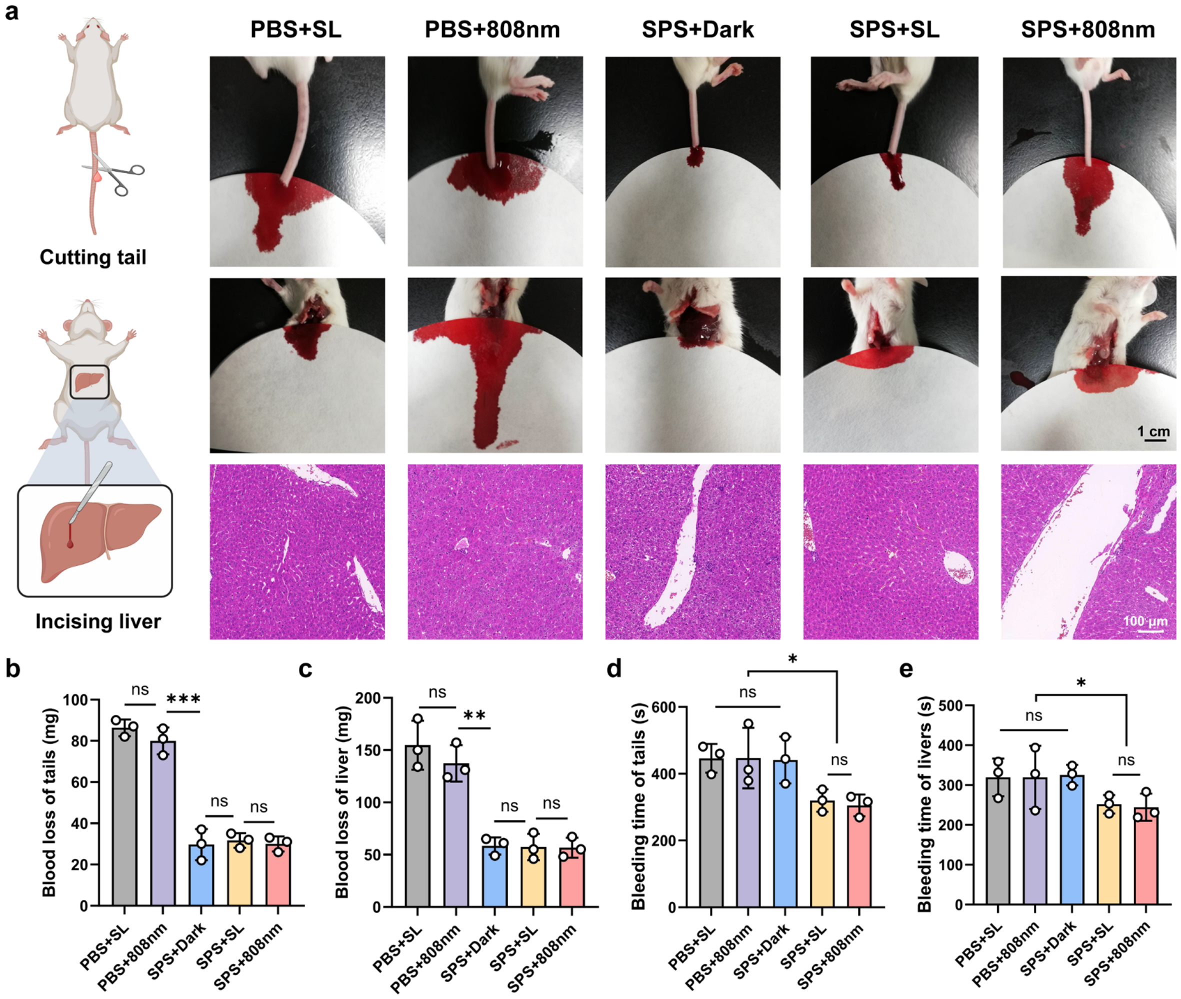

Fig. S22. Coagulation effect upon sunlight treatment. (a) Schematic representation of the tail vein bleeding model used to induce and control haemorrhage in the mouse liver, alongside photographic documentation of liver samples and quantification of blood loss following SPS treatment. Scale bars: 1 cm for whole-mouse images and 100 μm for H&E-stained liver sections. (b) Quantitative analysis of blood loss from the injured tail vein. (c) Quantitative analysis of blood loss from the hemorrhagic liver. (d) Quantitative analysis of bleeding duration in injured tails. (e) Quantitative analysis of bleeding time in injured livers. The results were obtained from the mice one week after treatment (n = 3 per group, *$p < 0.05$, **$p < 0.01$, ***$p < 0.001$, n.s.: not statistically significant, one-way ANOVA).

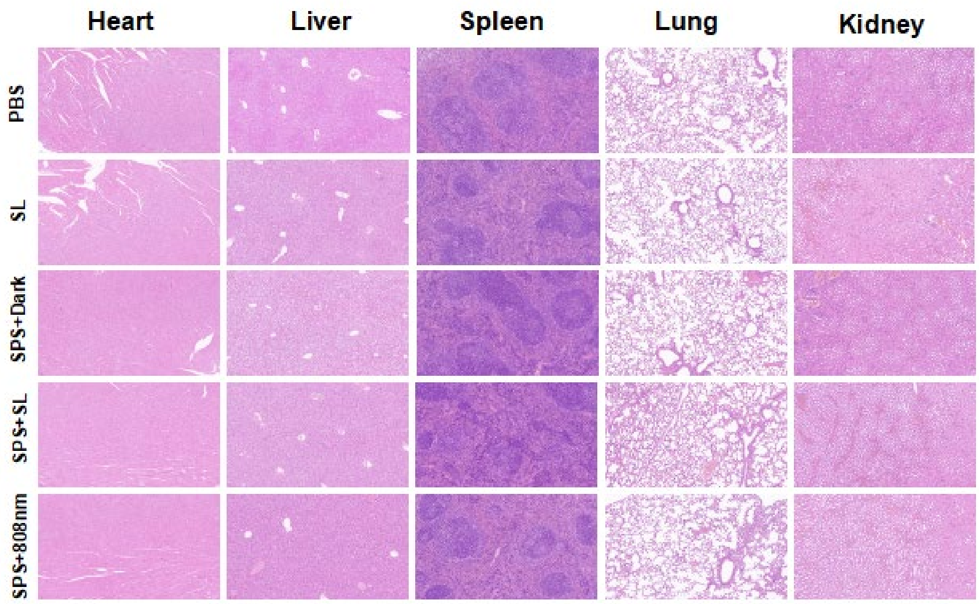

Fig. S23 HE staining of heart, liver, spleen, lung, and kidney in different groups.

a

No.	Inspection items	Results	References	Units
1	WBC	4.1	0.8-6.8	10^9/L
2	RBC	7.46	6.36-9.42	10^12/L
3	HGB	129	110-143	g/L
4	MCV	49.5	48.2-58.3	fL
5	PLT	407	450-1590	10^9/L
6	Lym#	3.87	0.7-5.7	10^9/L
7	Mid#	0.26	0-0.3	10^9/L
8	GR#	1.97	0.1-1.8	10^9/L
9	Lym%	63.5	55.8-90.6	%
10	Mid%	4.3	1.8-6	%
11	GR%	32.2	8.6-38.9	%
12	P_LCR	9.1	13-50	%
13	P_LCC	37	10-100	10^9/L
14	HCT	36.9	34.6-44.6	%
15	MCHC	350	302-353	g/L
16	RDW_CV	13.1	11-17	%
17	RDW_SD	52.9	33-50	fL
18	MCH	17.3	15.8-19	pg
19	MPV	9.1	3.8-6	fL
20	PCT	0.37	0.15-0.3	%
21	PDW	18.9	15-17	%

b

No.	Inpection items	Results	References	Units
1	WBC	10.15↑	0.8-6.8	10^9/L
2	RBC	6.82	6.36-9.42	10^12/L
3	HGB	114	110-143	g/L
4	MCV	48.9	48.2-58.3	fL
5	PLT	525	450-1590	10^9/L
6	Lym#	6.1↑	0.7-5.7	10^9/L
7	Mid#	0.33	0-0.3	10^9/L
8	GR#	1.72	0.1-1.8	10^9/L
9	Lym%	66.7	55.8-90.6	%
10	Mid%	5.3	1.8-6	%
11	GR%	28	8.6-38.9	%
12	P_LCR	11.3	13-50	%
13	P_LCC	59	10-100	10^9/L
14	HCT	33.4	34.6-44.6	%
15	MCHC	341	302-353	g/L
16	RDW_CV	10.2	11-17	%
17	RDW_SD	53.8	33-50	fL
18	MCH	16.7	15.8-19	pg
19	MPV	9	3.8-6	fL
20	PCT	0.473	0.15-0.3	%
21	PDW	20.7	15-17	%

c

No.	Inspectin items	Results	References	Units
1	WBC	5.87	0.8-6.8	10^9/L
2	RBC	7.24	6.36-9.42	10^12/L
3	HGB	118	110-143	g/L
4	MCV	49.8	48.2-58.3	fL
5	PLT	528	450-1590	10^9/L
6	Lym#	2.64	0.7-5.7	10^9/L
7	Mid#	0.43	0-0.3	10^9/L
8	GR#	2.8	0.1-1.8	10^9/L
9	Lym%	45	55.8-90.6	%
10	Mid%	7.3	1.8-6	%
11	GR%	47.7	8.6-38.9	%
12	P_LCR	28.6	13-50	%
13	P_LCC	151	10-100	10^9/L
14	HCT	36	34.6-44.6	%
15	MCHC	328	302-353	g/L
16	RDW_CV	10.6	11-17	%
17	RDW_SD	57.7	33-50	fL
18	MCH	16.3	15.8-19	pg
19	MPV	9	3.8-6	fL
20	PCT	0.475	0.15-0.3	%
21	PDW	18.4	15-17	%

Fig. S24 Routine blood test of (a) sham group, (b) PBS group, and (c) PDT group.

a

No.	Inspection items	Results	Units
1	ALT	18.6	U/L
2	AST	56.5	U/L
3	TBIL	1.2	umol/L
4	ALB	15.2	g/L
5	ALP	4	U/L
6	TG	0.42	mmol/L
7	LDL-C	0.14	mmol/L
8	HDL-C	0.25	mmol/L
9	GSP	986.2	umol/L
10	LDH	139	U/L
11	GLU	11.9	mmol/L
12	TP	28.7	g/L

b

No.	Inspection items	Results	Units
1	ALT	19.3	U/L
2	AST	52.6	U/L
3	TBIL	1.1	umol/L
4	ALB	15.4	g/L
5	ALP	2	U/L
6	TG	0.45	mmol/L
7	LDL-C	0.18	mmol/L
8	HDL-C	0.44	mmol/L
9	GSP	1061.2	umol/L
10	LDH	141	U/L
11	GLU	11.8	mmol/L
12	TP	25.5	g/L

c

No.	Inspection items	Results	Units
1	ALT	24.1	U/L
2	AST	54.6	U/L
3	TBIL	1.7	umol/L
4	ALB	15.3	g/L
5	ALP	7	U/L
6	TG	0.38	mmol/L
7	LDL-C	0.26	mmol/L
8	HDL-C	0.36	mmol/L
9	GSP	1004	umol/L
10	LDH	160	U/L
11	GLU	12.1	mmol/L
12	TP	28.9	g/L

Fig. S25 Blood biochemistry test of (a) sham group, (b) PBS group, and (c) PDT group.

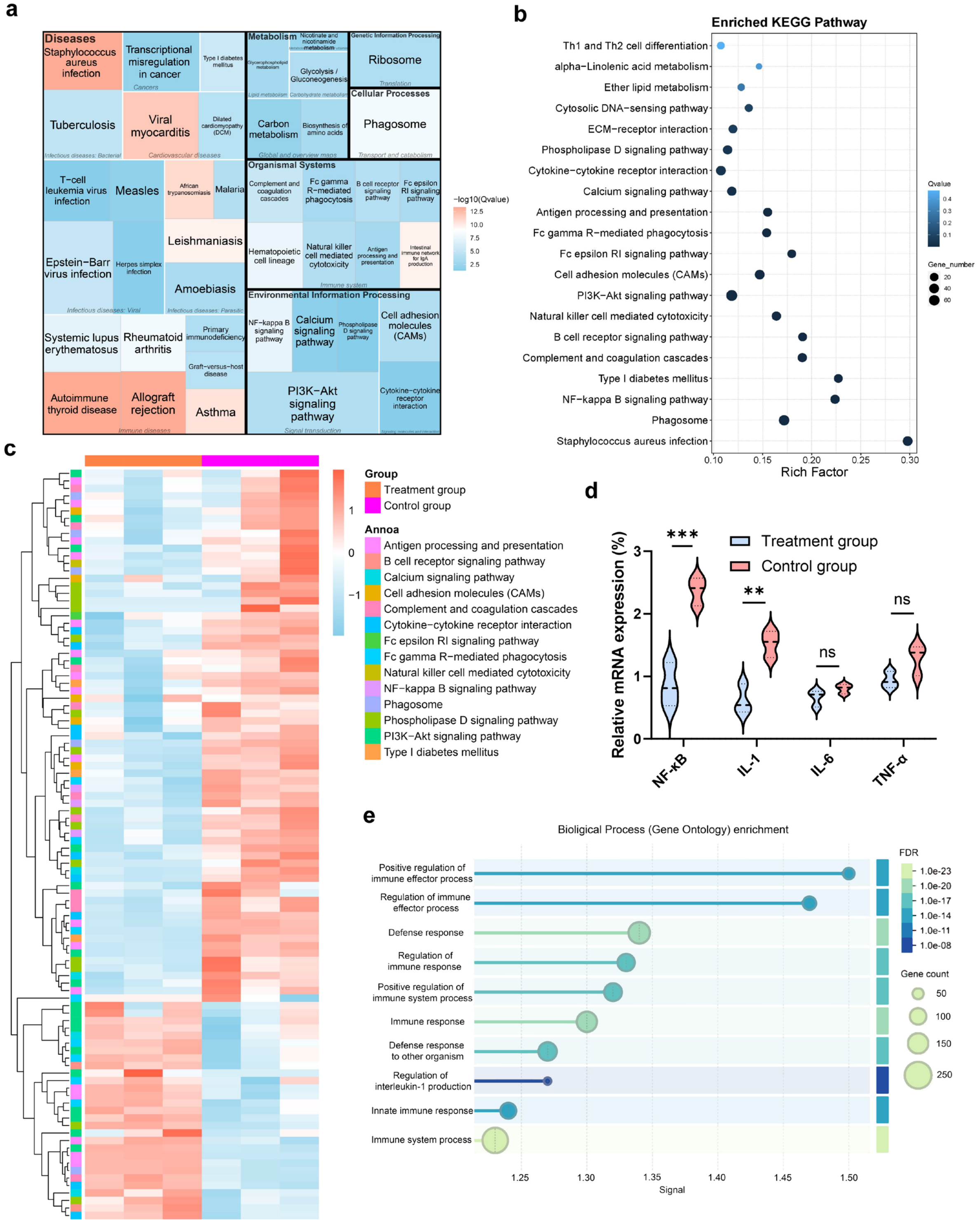

Fig. S26. Transcriptomics analysis. (a) Heatmap of differentially expressed pathways. (b) Bubble chart of enriched KEGG pathways. (c) Hierarchical clustering heatmap visualising the differential expression of genes across various tissue samples from the two groups (SPS with sunlight vs. PBS control). Green and red colours represent low and high gene expression abundance, respectively. Additionally, Voronoi plots highlight the top differentially expressed genes (DEGs) enriched in specific KEGG pathways. (d) Statistical analysis of relative mRNA expression of NF-κB, IL-1β, IL-6, and TNF-α, as assessed by qPCR. (e) Enrichment analysis of genes associated with different immune-related biological processes.

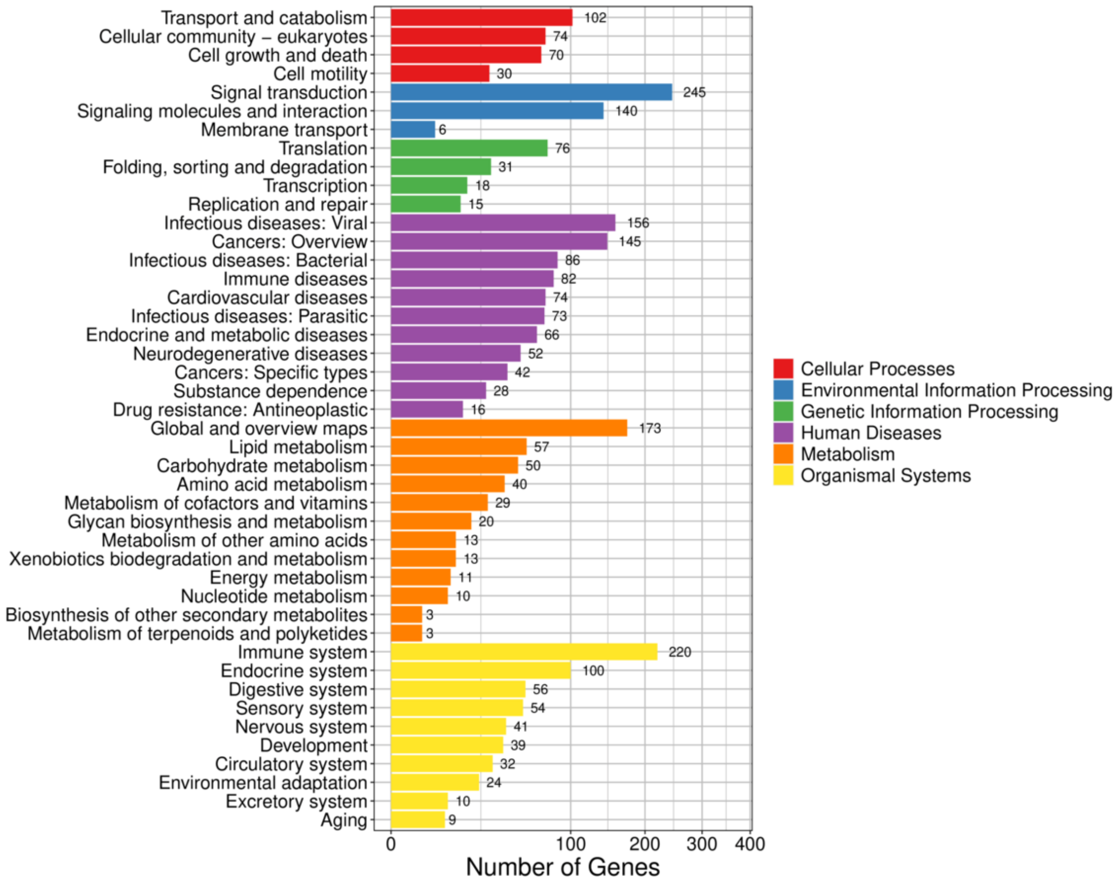

Fig. S27 Distribution of genes of selected pathways in transcriptome sequencing.

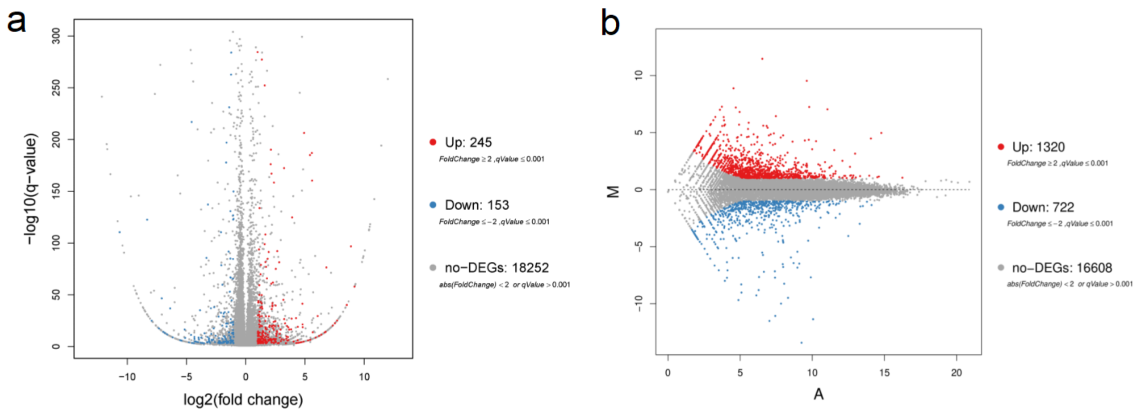

Fig. S28 (a) Up- and down-regulated volcano maps of treatment group genes compared with control group in transcriptome sequencing. (b) MA plot of treatment group genes compared with a control group using the DEG sequencing method.

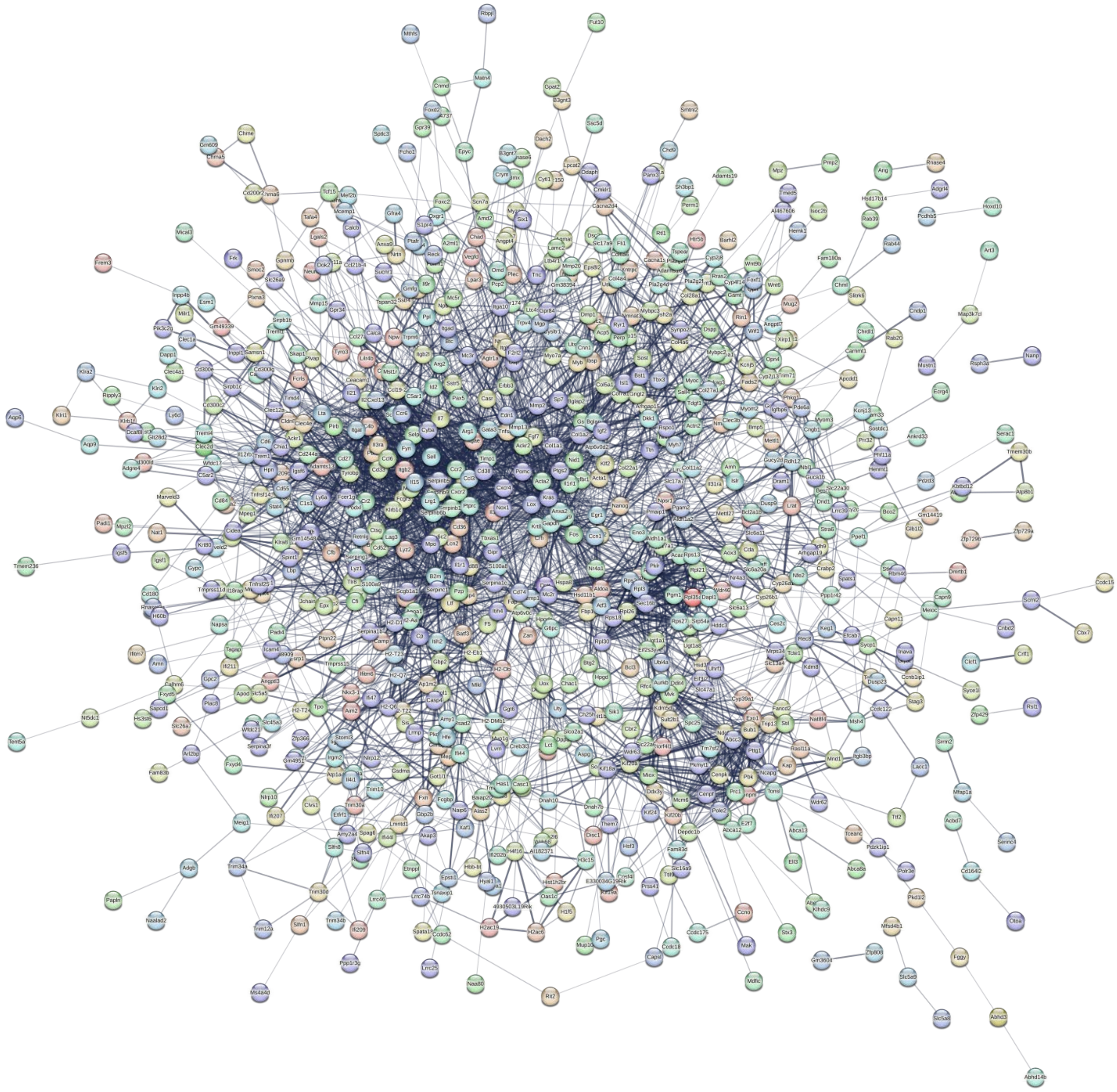

Fig. 29 Protein-protein interaction (PPI) network diagram. Threshold settings: pvalue or padj < 0.05.

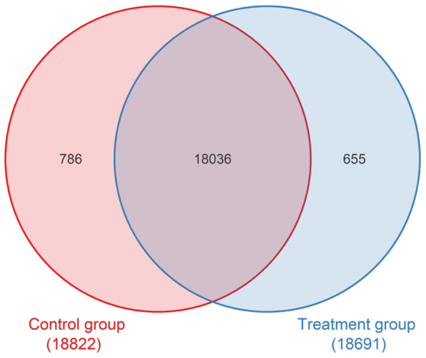

Fig. S30 Gene correlation Wayne diagram of control and treatment group.

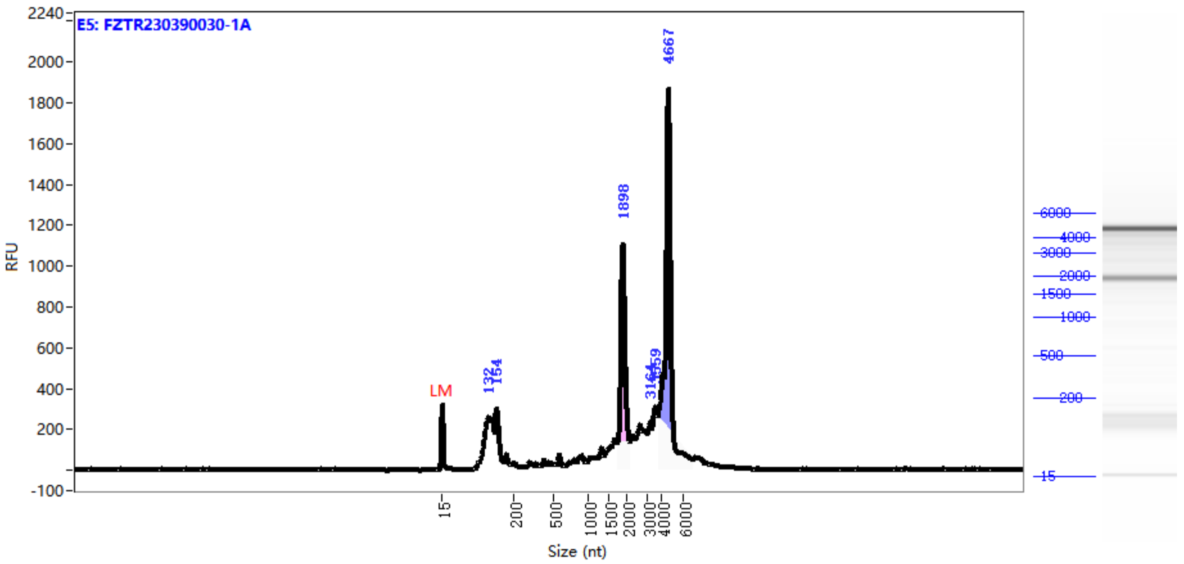

Fig. S31 Agilent 5400 peak plot of RNA (pass, smooth baseline). Horizontal coordinate: size (nt) distribution of fragments of the capillary sample in which the reaction is located. Vertical coordinate (RFU) value refers to the real-time fluorescence signal intensity of the sample d.
